# Enhancing the Heterologous Fructosyltransferase Activity of *Kluyveromyces lactis*: Developing a Scaled-Up Process and Abolishing Invertase by CRISPR/Cas9 Genome Editing

**DOI:** 10.3389/fbioe.2020.607507

**Published:** 2020-11-25

**Authors:** Jan Philipp Burghardt, Rong Fan, Markus Baas, Dustin Eckhardt, Doreen Gerlach, Peter Czermak

**Affiliations:** ^1^Institute of Bioprocess Engineering and Pharmaceutical Technology, University of Applied Sciences Mittelhessen, Giessen, Germany; ^2^Faculty of Biology and Chemistry, Justus Liebig University, Giessen, Germany; ^3^Department of Bioresources, Fraunhofer Institute for Molecular Biology and Applied Ecology IME, Giessen, Germany

**Keywords:** chemically-defined fermentation medium, CRISPR/Cas9, fructosyltransferase (FFT), prebiotic, *Kluyveromyces lactis* GG799, fructo-oligosaccharides (FOS), high-cell-density cultivation

## Abstract

The enzymatic production of prebiotic fructo-oligosaccharides (FOS) from sucrose involves fructosyltransferases (FFTs) and invertases, both of which catalyze forward (transferase) and reverse (hydrolysis) reactions. FOS yields can therefore be increased by favoring the forward reaction. We investigated process conditions that favored transferase activity in the yeast strain *Kluyveromyces lactis* GG799, which expresses a native invertase and a heterologous FFT from *Aspergillus terreus*. To maximize transferase activity while minimizing native invertase activity in a scaled-up process, we evaluated two reactor systems in terms of oxygen input capacity in relation to the cell dry weight. In the 0.5-L reactor, we found that galactose was superior to lactose for the induction of the *LAC4* promoter, and we optimized the induction time and induction to carbon source ratio using a response surface model. Based on the critical parameter of oxygen supply, we scaled up the process to 7 L using geometric similarity and a higher oxygen transport rate, which boosted the transferase activity by 159%. To favor the forward reaction even more, we deleted the native invertase gene by CRISPR/Cas9 genome editing and compared the Δ*Inv* mutant to the original production strain in batch and fed-batch reactions. In fed-batch mode, we found that the Δ*Inv* mutant increased the transferase activity by a further 66.9%. The enhanced mutant strain therefore provides the basis for a highly efficient and scalable fed-batch process for the production of FOS.

## Introduction

Fructo-oligosaccharides (FOS) are fructose oligomers in which the fructose units are linked by β(2 → 1) glycosidic bonds, often with a terminal α(2 → 1) linked glucose residue. This configuration of glycosidic bonds means FOS cannot be digested by humans, but they stimulate the growth of bifidobacteria and lactobacilli in the gastrointestinal tract, thus conferring prebiotic properties (Figueiredo, 2020). FOS have 30–50% of the sweetening power of sucrose (Florowska et al., 2016) and are therefore used as sugar replacements in food and beverage products (Sánchez-Martínez et al., 2020). Furthermore, prebiotic FOS fibers can be used to replace the fat content of fermented sausage products due to the similar texture (Bis-Souza et al., 2020). Approximately 10% of natural sweeteners are based on FOS (Kumar et al., 2018). These molecules are naturally produced by plants, bacteria and fungi, but their low native concentration makes extraction impractical (Fujita et al., 1990; van den Ende and van Laere, 1996; Ning et al., 2010). In contrast, enzymatic production can achieve high yields and, unlike chemical synthesis, is regioselective.

Enzymes that catalyze the synthesis of FOS are known as β-fructofuranosidases (or invertases) and fructosyltransferases (FFTs). Both enzymes catalyze the hydrolysis of sucrose and the synthesis of FOS. The higher the underlying transferase activity, the higher the potential FOS yield. Therefore, the combination of high transferase and low hydrolysis activity is beneficial for industrial-scale production. Furthermore, higher transferase activity favors the production of longer-chain FOS, which are more stable in a low-pH environment (Blecker et al., 2002; Vega and Zuniga-Hansen, 2015). The pH stability of FOS is particularly important because the exposure of short-chain FOS to gastric acid can lead to the release of fructose units (Nilsson and Björck, 1988). The length of the fructosyl chain also determines where FOS are fermented by intestinal bacteria, with shorter-chain FOS broken down in the proximal part of the colon and longer-chain FOS broken down more distally (van Loo, 2004). The enzymatic synthesis of FOS is advantageous because the chain length can easily be defined by controlling the reaction time and conditions.

The industrial-scale production of FOS requires the expression of microbial enzymes (Ganaie and Gupta, 2014), which can be applied as whole-cell catalysts, crude enzyme extracts, or purified enzymes immobilized on a membrane or other carrier (Sánchez-Martínez et al., 2020). Given that FOS are intended for the food and feed sector, the enzymes used for this purpose must originate from safe expression systems such as the yeast *Kluyveromyces lactis*, which has “generally recognized as safe” (GRAS) status for the production of β-galactosidase and therefore meets safety requirements. However, native *K. lactis* secretes invertase, favoring the hydrolysis of sucrose during the production of FOS.

To improve the yield of FOS, we established a scalable process in laboratory scale, which favors the heterologous transferase activity in the cell-free fermentation broth. In the first part of the study, we focused on a process engineering approach by screening suitable inducers, induction ratios, and induction times (Panuwatsuk and Da Silva, 2003). We then scaled up the process from 0.5 to 7 L to increase the cell dry weight (CDW), basing our strategy on two previous reports, the first showing that total aerobic conditions increase the biomass of *K. lactis* cultures at a dissolved oxygen (DO) concentration of 40–60% (Blondeau et al., 1994), and the second showing that glucose in the fermentation medium represses the *K. lactis* native invertase gene (Burghardt et al., 2019a). Accordingly, we characterized the oxygen supply in two reactor systems at different scales, and scaled up the process from 0.5 to 7 L by maintaining the oxygen transfer rate and thus ensuring that enzymatic transferase activity and process parameters such as CDW, DO concentration and protein concentration were comparable. In the second part of the study, we developed a fed-batch process at the 7-L scale under aerobic conditions, revealing a limitation that could not be addressed by process engineering. We therefore used the CRISPR/Cas9 system to inactivate the native *K. lactis* invertase gene and tested the biomass yield and transferase activity against the original production strain in batch and fed-batch fermentations.

## Materials and Methods

### Strains and Plasmids

#### *K. lactis* Production Mutant

The *K. lactis* GG799 production host was generated as previously described (Spohner and Czermak, 2016) by introducing an FFT gene from *A. terreus* NIH2624 into the *K. lactis* GG799 wild-type strain from the *K. lactis* Protein Expression Kit (New England Biolabs, Frankfurt, Germany).

#### CRISPR/Cas9 Plasmid and Invertase Deletion

The CRISPR/Cas9 system was used to inactivate the invertase gene (GenBank: AF079370.1) in the *K. lactis* GG799 mutant. The CRISPR/Cas9 plasmid pUDP002 was a gift from Jean-Marc Daran (RRID:Addgene_103872) and in addition to the *cas9* gene contains a selectable marker conferring resistance to hygromycin B and two BsaI restriction sites for the insertion of guide RNA sequences. The 20-bp protospacer sequence for *K. lactis* invertase was designed using the CRISPR gRNA design tool (https://www.dna20.com/eCommerce/cas9/input) and synthesized with terminal BsaI sites by Thermo Fisher Scientific (Dreieich, Germany). The pUDP002 vector and insert were digested with BsaI, ligated, and introduced into *K. lactis* GG799 along with single-stranded salmon sperm DNA and double-stranded repair DNA as previously described (Lin-Cereghino et al., 2005). The repair DNA matched the first 75 bp at the 5′ flank of the invertase gene (TAA TGT TTG AGA TCA CAC TTC AAA CAT GTT CAC AAT AAT TGA TGG AAA CAC ACA ATT GGA ATC AAA AGG GTA TCG) and the first 75 bp at the 3′ flank (ATT TTA TTT CGC TTT GAT GAG TGT GCT GCT TTA AGT CAA GGA GAT GGC AAG GAA ATG ACA CAT ACA TAT TGA TAT) and was included to promote homologous recombination at the double-strand break introduced by Cas9, thus leading to the complete deletion of the invertase locus (Δ*Inv*). This was confirmed by selection on YPD agar plates supplemented with 100 μM hygromycin B followed by sequencing across the deleted locus. Briefly, genomic DNA was isolated, purified and resuspended (10 mM Tris-HCl pH 7.5, 1 mM EDTA), and 1 ng was amplified with Q5 Hot Start High-Fidelity DNA polymerase in Q5 High-Fidelity 2× Master Mix (New England Biolabs) in a PEQLAB thermocycler (Erlangen, Germany) using appropriate primers (10 μM) synthesized by Sigma-Aldrich (Taufkirchen, Germany). The reaction was heated to 98°C for 30 s followed by 30 cycles of 98°C for 15 s, 57°C for 20 s, and 72°C for 40 s, and a final extension step at 72°C for 2 min. PCR products were isolated using the GeneJET DNA Purification Kit (Thermo Fisher Scientific) and sequenced by Microsynth Seqlab (Göttingen, Germany).

### Fermentation Medium

The fermentation medium was based on FF22 medium for yeast (Stratton et al., 1998; Burghardt et al., 2018). One liter of the fermentation medium contained 30 g glucose and 7.5 g galactose as a carbon source, 5.161 g Na_3_C_6_H_5_O_7_, 1 g CaSO_4_, 8.6 g K_2_SO_4_, 1 g MgSO_4_, and 10 g (NH_4_)_2_SO_4_ as basal salts, 0.92 g K_2_HPO_4_ and 13.54 g KH_2_PO_4_ as buffer components, 1.72·10^−4^ g H_3_BO_3_, 1.72·10^−2^ g CuSO_4_, 2.58·10^−2^ g MnSO_4_, 0.1897 g FeSO_4_, 8.6 μL H_2_SO_4_, 1.72·10^−3^ g NaMoO_4_, 4.3·10^−3^ g CoCl_2_, 6.02·10^−2^ g ZnCl_2_, and 6.88·10^−3^ g NaI as micronutrients, and 0.4·10^−3^ g biotin, 1.025·10^−3^ g pantothenic acid, 1.025·10^−3^ g nicotinic acid, and 1.025·10^−3^ g pyridoxine as vitamins. All chemical reagents were purchased from Sigma-Aldrich or Carl Roth (Karlsruhe, Germany).

### Analytical Methods

#### Activity Assay and UHPLC Analysis

The activity of the cell-free crude enzyme solution was determined as previously reported (Burghardt et al., 2019a).

#### Protein Analysis

The protein concentration in cell-free supernatants was determined using the Bradford Assay (AppliChem, Darmstadt, Germany). Protein samples were mixed with Bradford reagent in microtiter plates and the absorbance was measured at 465 nm and 595 nm. A calibration line was established at concentrations of 0–200 μg L^−1^ bovine serum albumin (BSA). The protein concentration was calculated from the calibration line.

#### Optical Density and CDW

Growth in shaking flasks and reactors was monitored by measuring the optical density at 600 nm (Δ*OD*_600_) with a BioSpectrometer Kinetic (Eppendorf, Hamburg, Germany). If the measurements exceeded *OD*_600_ = 0.6, the samples and the blank were diluted with 0.9% (w/v) NaCl and the corresponding Δ*OD*_600_ measurement was multiplied by the dilution factor. The CDW was determined at the end of the fermentation. The empty mass of a 2-mL microfuge tube was determined after drying (65°C, 24 h). We then centrifuged 2 mL of culture broth at 16,000 × g for 1 min at room temperature, washed the cell pellet by resuspending it in 2 mL 0.9% (w/v) NaCl, centrifuged the suspension as above, dried the pellet at 65°C for 24 h and determined the mass again. The CDW was recorded as the difference between the empty and full mass of the microfuge tube.

### Bioreactor Cultivation

**Figure 1:**
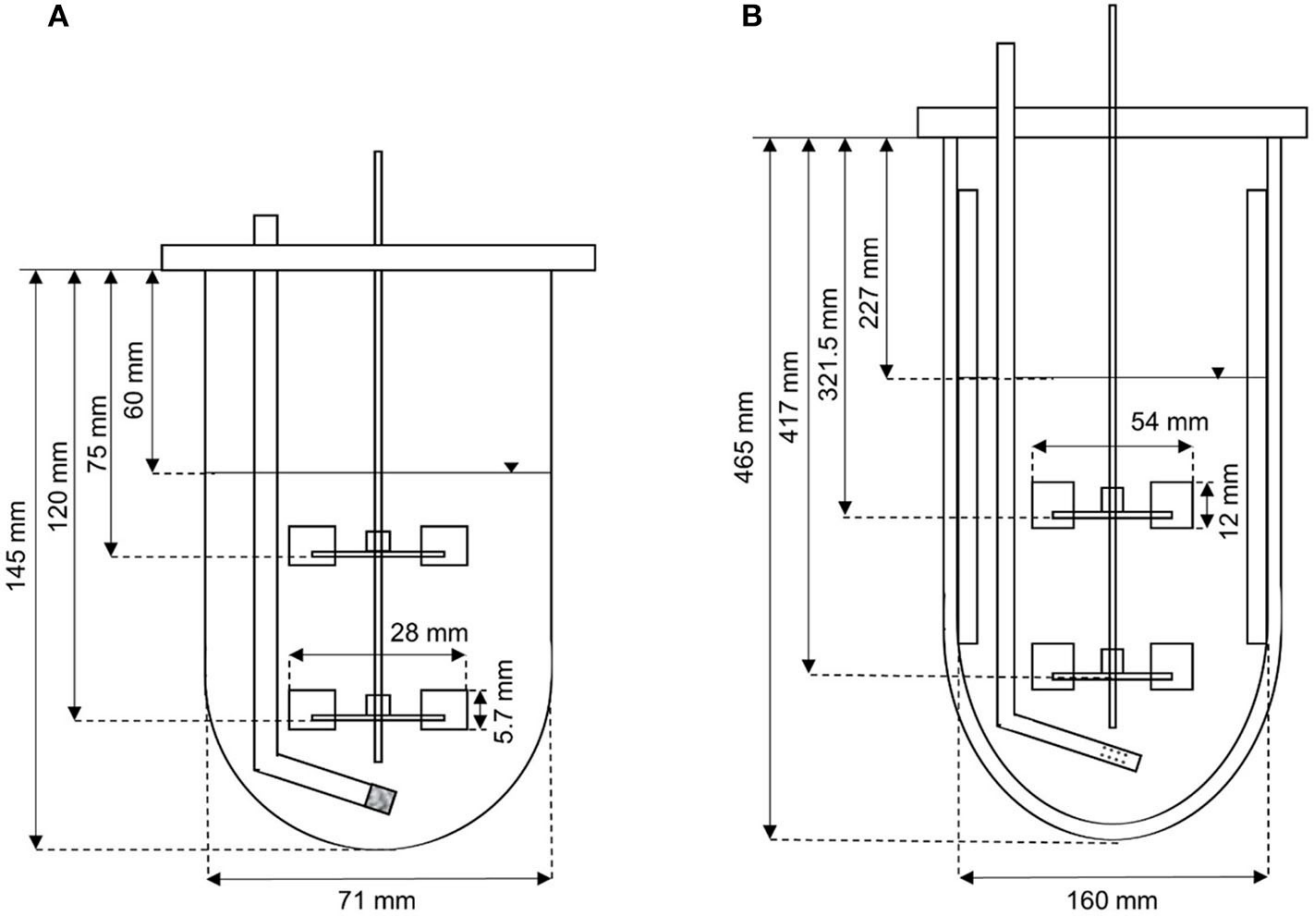
Setup of **(A)** the Applikon MiniBio500 system, and **(B)** the Infors Labfors3 system for the fermentation of *K. lactis*.

Two different reactor scales were used for process development. The Applikon MiniBio500 system with a total volume of 500 mL was operated at a working volume of 300 mL. The Infors Labfors3 system with a total volume of 7 L was operated at a working volume of 3.43 L. Characterization of the bioreactors (Figure 1) was necessary to scale up the process based on fixed conditions (often dimensionless numbers/critical parameters describing physical states or processes) to ensure physical similarity by maintaining the values, thus avoiding transport limitations on a larger scale. The geometric similarity of the two stirred tanks allowed a simple and comparable reactor design, hence scale-up was limited to the stirrer peak speed and volumetric power input P/V (Equation 1).

(1) $$\begin{array}{l} \frac{D_{2}}{D_{1}}\;={\left(\frac{V_{2}}{V_{1}}\right)}^{\frac{1}{3}} \end{array}$$

where *D*_1_ and *D*_2_ are the small-scale and large-scale vessel diameters, respectively, and *V*_1_ and *V*_2_ are the corresponding liquid working volumes. This equation applies under the following conditions: $\frac{d}{D}=0.3-0.45;\;\frac{H_{L}}{D}<2;N=2\vee \;3$.

#### Measuring the K_L_a and Estimation of Oxygen Solubility

The volume-related mass transfer coefficient (K_L_a) was determined during cultivation by dynamic outgassing, and an online K_L_a value was determined by exhaust gas analysis (Bluesens Gas Sensor GmbH, Herten, Germany). Dynamic outgassing with microorganisms (Wise, 1951) was carried out as previously described (Bandyopadhyay et al., 1967) using a Clarke electrode (0.5-L system) or optode (7-L system). Measurements were taken in the Δ*OD*_600_ range 20–40 and K_L_a was calculated as shown in Equation (2) for logarithmic applications. To ensure the K_L_a is correct, the response time of the Clarke electrodes must be shorter than the reciprocal of the K_L_a value. Because the volumetric mass transfer coefficient changes during the course of fermentation, a distinction is made between K_L_a in the presence of microorganisms and k_L_a in their absence.

(2) $$\begin{array}{l} K_{L}a\;=\;\frac{\ln\left(c_{t}-c_{0}\right)}{t} \end{array}$$

The exhaust gas analyzer took into account the liquid volume, gas flow, and the CO_2_ and O_2_ concentrations in the supply and exhaust air (Meiners, 1990). This was calculated online using Equation (3).

(3) $$\begin{array}{l} OTR_{online}\\ =\;\left(\frac{F_{in}}{V_{fluid.}V_{M}}\right).\left(c_{O_{2},in}-c_{O_{2},out}\frac{\left(1-c_{O_{2},in}-c_{CO_{2},in}\right)}{\left(1-C_{O_{2},out}-c_{CO_{2},out}\right)}\right) \end{array}$$

The maximum gas flow rate *Q*_max_ and thus maximum volume flow $\dot{V}$ can be expressed for a stirred tank by means of the Froude number (Equation 4). This correlation is given in the range 0.1 < Fr < 2 (Zlokarnik, 1991). It describes the maximum permissible gas volume flow for a stirrer speed at which no flooding of the stirrer occurs.

(4) $$Q_{\max}\;=\;\frac{\dot{\text{V}}}{{\text{nd}}_{\text{i}}^{\text{3}}}\;=\;0.19\times {\text{Fr}}^{0.75}$$

The medium components are weighted according to their concentration and valence (which together correspond to the ionic strength) and the salting out coefficient to determine the solubility (Equation 5) or the Bunsen coefficient (Equation 6). To infer the maximum solubility from the logarithmic quotient of the Bunsen coefficient (Equation 6), the Bunsen coefficient for pure water (α_0_) must be calculated (Equation 7) (Schumpe et al., 1982; Prausnitz et al., 1986). Subsequently, by defining the K_L_a, the OTR can be calculated and the scale transfer can be achieved.

(5) $$\log_{10}\left(\frac{{\text{C}}_{\text{AL0}}^{*}}{{\text{C}}_{\text{AL}}^{*}}\right)\;=\;0.5\sum {\;}_{\text{i}}^{\;}{\text{H}}_{\text{i}}{\text{z}}_{\text{i}}^{\text{2}}{\text{C}}_{\text{iL}}+\sum {\;}_{\text{j}}^{\;}{\text{K}}_{\text{j}}{\text{C}}_{\text{jL}}$$

where $c_{AL0}^{*}$ is the oxygen solubility without soluble components, $c_{AL}^{*}$ is the oxygen solubility, H_i_ is a constant for the ionic component i, z_i_ is the valence of the ionic component i, *c*_*iL*_is the concentration of ionic component i in the liquid, *K*_*j*_ is a constant for non-ionic component j, and *c*_*jL*_ is the concentration of the non-ionic component j in the liquid (Prausnitz et al., 1986).

(6) $$\begin{array}{l} \log\left(\frac{\alpha_{0}}{\alpha }\right)\;=\;\sum_{\text{i}}\left({\text{H}}_{\text{i}}{\text{I}}_{i}\right) \end{array}$$

(7) $$\begin{array}{l} \alpha_{0}\;=\;4.9\;\times \;10^{-2}\;-\;1.335\;\times {\;10}^{-3}\;\times \text{ T }+\;2.759\;\\ \;\times {\text{ T}}^{2}\;-\;3.235\;\times {\;10}^{-7}\times {\text{T}}^{3}\;+\;1.614\;\times \;10^{-9}\\ \;\times {\;T}^{4} \end{array}$$

To estimate the theoretically determined solubility of oxygen in the medium, the solubility was determined experimentally by two different methods. In the first approach, we adapted the method of Vendruscolo et al. (2012). Briefly, the method is based on the enzymatic conversion of hydrogen peroxide to oxygen and water catalyzed by a catalase. The oxygen is measured using a DO probe in the fermenter (Käppeli and Fiechter, 1981; Vendruscolo et al., 2012). The method was modified such that the head space of the fermenter was gassed with nitrogen during catalysis to minimize oxygen input from the air. In the second approach, the oxygen saturation during a fermentation with a known biomass and oxygen consumption rate was determined as previously described (Czermak et al., 2009). The calculations are provided in the Supplementary Material.

### Optimization of the Carbon Source

The *LAC4* promoter must be induced for high protein expression throughout the fermentation process, and this can be achieved by varying the inducer strength. An upstream growth phase can be advantageous for heterologous protein expression, so we investigated an expression strategy.

#### Shaking-Flask Cultivation

The selection of a carbon source was performed in FM22 medium in shaking flasks with 20 g L^−1^ each of the corresponding carbon source (glucose, galactose, and lactose). The two sugars lactose and galactose, which induce the LAC4 promoter, and glucose as a further carbon source were selected for one-factor-at-a-time experiments. However, the LAC4 promoter is a leaking promoter and therefore also shows a certain protein expression in the absence of inducers. The cryocultures used for inoculation were centrifuged at 4°C and resuspended in 0.9% NaCl solution, so that no falsification of the sugar concentration occurred. The shaking flasks were inoculated with the washed cryocultures at Δ*OD*_600_ = 0.1–0.2. Cells were grown in flasks at 30°C shaking at 150 rpm, with three baffles for increased oxygen supply. The volume was 500 mL with a working volume of 10%. The shake flasks were harvested in the stationary growth phase, cell-dry weight, yield coefficients, and volume activity were determined to compare the influence of each carbon source.

#### Inductor Comparison in Reactor Scale

Reactor-scale experiments to compare the influence of lactose and galactose for induction in 500 mL scale were conducted using in 300 mL of the FM22 medium supplemented with 30 g L^−1^ glucose as carbon source and 7 g L^−1^ of galactose or lactose as inductor. The *K. lactis* strain was cultivated in the adjusted FM22 medium at 30°C. The pH maintained at 6 using 25% (v/v) ammonia. The DO concentration in the medium was controlled in a cascade of stirrer speed, aeration rate, and oxygen partial pressure. To avoid foaming, we added 0.02% (v/v) Struktol J673A (Schill & Seilacher, Hamburg, Germany) to the medium before the start of fermentation. The reactor was inoculated with a cryostock or pre-culture at Δ*OD*_600_ = 0.1–0.2 and harvested during the stationary phase. The temperature was controlled using a Peltier element and air was introduced into the system via a porous sparger. The temperature was controlled using a double jacket tempered by water. Air or an air/oxygen mixture was introduced into the system via a gassing tube. The reactors were harvested in the stationary growth phase analogous to the shaking bottle experiments. As parameters the obtained cell-dry weight and transfer activity were compared.

#### Optimization of Induction

In a publication by Panuwatsuk and Da Silva (2003) the influence of the initial glucose to galactose ratio on the cell-dry weight and beta-galactosidase activity was investigated in a *K. lactis* strain with one-factor-at-a-time experiments. Following this experimental setup, we performed a D-optimal design to optimize the transfer activity of the FFT exposed in this work. As factors of the experimental space, the point of induction in terms of optical density (ΔOD_600_) in a range of 0.01 and 120 and the induction influence of the galactose:glucose ratio (within the limits 0.05–0.45) was investigated. When varying the galactose induction strength as a ratio, a constant starting concentration of 30 g L^−1^ glucose was applied. The corresponding amount of galactose was added at the respective time of induction within the limits of 0.1–120 Δ*OD*_600_. The experimental setup was analogous to the 500 mL scale experiments of the inductor compare comparison.

## Results

### Carbon Source Screening

We supplemented FM22 medium in shaking flasks with 20 g L^−1^ of each carbon source (glucose, lactose, or galactose). The cryocultures used for inoculation were centrifuged at 4°C and resuspended in 0.9% NaCl to ensure they did not affect the overall sugar concentration. The cells were harvested during the stationary phase and we determined the CDW as well as the transferase activity and pH of the cell-free broth. We were particularly interested in the CDW and biomass yield because the leaky *LAC4* promoter is only induced by galactose and lactose. Growth on glucose achieved the highest CDW of 9.7 ± 0.2 g L^−1^ with a biomass yield (Y_x/s_) of 0.49, followed by galactose (9.0 ± 0.1 g L^−1^; Y_x/s_ = 0.45) and lactose (8.5 ± 0.2 g L^−1^; Y_x/s_ = 0.43). The highest transferase activity of 10.8 ± 0.7 U mL^−1^ was detected in the supernatant of cultures supplemented with galactose. Given the ability of glucose to inhibit native *K. lactis* invertase (Georis et al., 1999) and to outperform the other carbon sources in terms of CDW and biomass yield, we selected glucose as the carbon source.

### Reactor Cultivations

#### Theoretical Design of Reactor Systems Using Kinetic Data for *K. lactis*

Reactor systems should be designed to ensure that sufficient oxygen is supplied for an aerobic process, which requires knowledge of the fluid dynamic characteristics of the reactor, growth and substrate uptake kinetics, and the metabolic pathways of the cultivated strain. The maximum oxygen input can be calculated by empirical approximations (Henzler, 1982; van't Riet, 1983). For the Infors reactor, the maximum k_L_a was 3.8 and 20.0 min^−1^ for coalescent and non-coalescent systems, respectively (van't Riet, 1983). With a minimum DO of 50%, an oxygen solubility $c_{L}^{*}\;$of 6.45 at 30°C and atmospheric pressure, we calculated corresponding OTRs of 0.59 and 3.87 g L^−1^ h^−1^, respectively (Table 1). To prevent flooding at high gas volume flows, the calculation of the maximum gas flow rate (Equation 4) was applied to the 0.5 L reactor-scale (Equation 8) and 7 L scale (Equation 9) to calculate the minimum stirrer speed:

(8) $$\begin{array}{l} Q_{\text{max},\;0.5\text{ L}}=27\text{ VVM},\text{ at }1,400\text{ rpm} \end{array}$$

(9) $$\begin{array}{l} Q_{\text{max},\;7\text{ L}}=6.8\text{ VVM},\text{ at }800\text{ rpm} \end{array}$$

**Table 1:** Reactor designs for coalescent and non-coalescent systems with regard to k_L_a, OTR, maximum substrate and biomass concentration (CDW), and uptake rate under the condition DO > 50%.

	**Labfors3**		**MiniBio**	
	**Coalescent**	**Non-coalescent**	**Coalescent**	**Non-coalescent**
^a^k_L_a (min^−1^)	3.8^α^	20.0^β^	3.9^γ^	25.5^δ^
OTR (g L^−1^ h^−1^)	0.59	3.87	0.65	4.94
^b^c_s, max_ (g L^−1^)	6.5	42.5	7.2	54.3
^b^CDW_max_ (g L^−1^)	3.12	20.4	3.46	26.1
r_s, max_ (g L^−1^ h^−1^)	1.15	7.55	1.28	9.64

a *Maximum k_L_a for aeration rates of ^α^2.2 vvm, ^β^1 vvm, ^γ^3 vvm, ^δ^2 vvm*.

b *Determined assuming μ = μ_max_*.

To calculate the oxygen demand, a consumption of 6 mol O_2_ per mol glucose/galactose was assumed (Benveniste et al., 2018). Using the yield coefficients Y_x/glucose_ = 0.49 and Y_x/galactose_ = 0.45, the maximum biomass at a given carbon source concentration can be calculated, ignoring the influence of heterologous expression on growth. The consumption rate r was determined for the two sugars from the growth rate and yield coefficient. This was based on a literature value of μ = 0.37 h^−1^ for defined media (Dickson and Markin, 1980) as shown in Equation (10).

(10) $$\begin{array}{l} \text{OTR}={\bar{\text{Y}}}_{\text{x/s}}\;\times \;({\text{c}}_{\text{Glucose}}\text{ + }{\text{c}}_{\text{Galactose}})\;\times \;({\text{r}}_{\text{Glucose}}\text{ + }{\text{r}}_{\text{Galactose}})\\ \;\times \text{ 6 mol }{\text{O}}_{\text{2}}\;\cdot \text{ 32}\frac{\text{g}}{\text{mol}} \end{array}$$

By isolating the carbon source concentration from Equation (3), we calculated a maximum substrate concentration (glucose and galactose) of 54.3 g L^−1^, and thus a CDW of 26.1 g L^−1^, in the MiniBio reactor. For the Labfors3 system at atmospheric pressure and DO > 50%, the maximum concentration of substrate is 42.5 g L^−1^ achieving a CDW of 20.4 g L^−1^.

#### Measuring the K_L_a

The K_L_a was measured using the dynamic outgassing method in the MiniBio and Labfors3 systems (Cho and Wang, 1990). The stirrer speed and aeration rate at the beginning and end of fermentation in the MiniBio system were set for measurement so that K_L_a values of 1.7 and 2.15 were achieved at the beginning and end of fermentation, respectively (Table 2). In the Labfors3 system, this requires 800 rpm and 1 vvm as starting parameters and 1,200 pm and 1.5 vvm as ending parameters. We also investigated the K_L_a in the Labfors3 system in the ranges 0.5–1.5 vvm and 800–1,200 rpm using the response surface method. The highest coefficient of 2.3 was achieved at 1.5 vvm and 1,200 rpm. The lowest coefficient of 1.5 was observed at 1.5 vvm and 800 rpm.

**Table 2:** Comparison of stirrer speed and gassing rate parameters for setting the K_L_a of the MiniBio and Labfors3 systems.

**K_**L**_a (min^**−1**^)**	**System**	
	**Parameter_**MiniBio**_**	**Parameter_**Labfors3**_**
1.7/1.7	1,400 rpm, 1 vvm	800 rpm, 1 vvm
2.15/2.1	1,800 rpm, 1 vvm	1,200 rpm, 1 vvm

The critical DO concentration is an important parameter in process technology because it indicates the DO concentration that supports respiratory metabolism of the carbon source. At higher DO values growth becomes oxygen independent. This parameter was determined by dynamic outgassing (Cho and Wang, 1990). At a DO of 25%, the decrease was no longer linear, representing the critical DO concentration of 1.62 mg L^−1^. The maximum *K. lactis* biomass in defined MBG20 medium was achieved at a DO concentration of 40–60% (Blondeau et al., 1994), so we adjusted the DO of the bioreactor cultures to 50% if not stated otherwise. Given the growth-associated heterologous expression driven by the *LAC4* promoter, the biomass produced is of great importance as a parameter for process development (Sakhtah et al., 2019).

#### Comparison of Galactose and Lactose as Inducers in the 0.5-L Reactor

The influence of galactose and lactose as inducers of FFT expression in the 0.5-L reactor was investigated under standard conditions. Initially, we compared 7.5 g L^−1^ of lactose and galactose, and found that with the same CDW (21.2 g L^−1^) the galactose-induced process achieved a higher activity (166 U mL^−1^) than lactose (92 U mL^−1^) after 20 min. The transferase ratio of both approaches was 85%, so galactose was selected as the inducer for subsequent experiments.

#### Influence of Inducer Concentration and Induction Time

**Figure 2:**
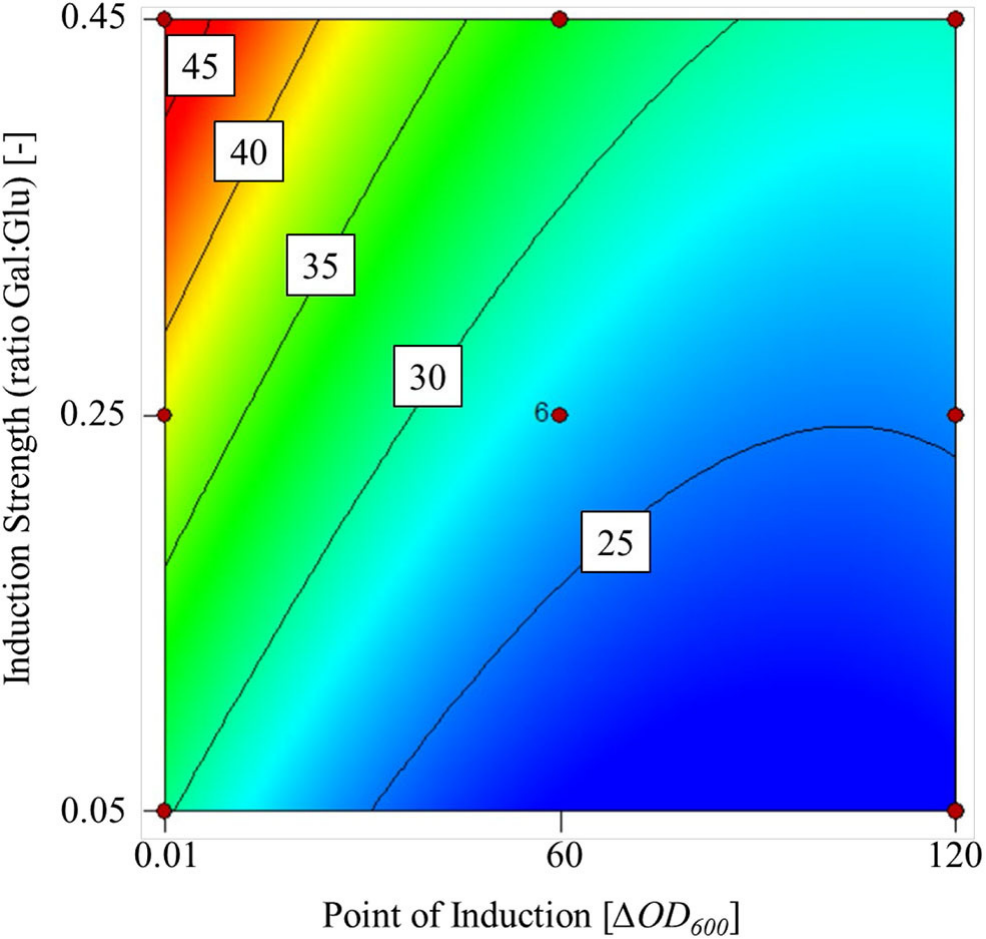
Contour plot of the mathematical model used to predict transferase activity based on two factors: point of induction (Δ*OD*_600_) and induction strength (ratio Gal:Glu) within the experimental limits.

Higher galactose concentrations increased the transferase activity in the supernatant at the end of fermentation, but a delayed induction had a negative effect (Figure 2). The maximum transferase activity within the experimental limits was 48 U mL L^−1^ and the lowest was 22 U mL L^−1^.

We found that the point of induction and induction strength had a significant influence on the transferase activity, *p* = 0.0012 and *p* = 0.0040, respectively. Analysis of variance (ANOVA) confirmed that the cubic model fit was significant (*p* = 0.0011) with a non-significant lack of fit (*p* = 0.1759) at a 5% significance level. The *R*^2^ value of the fit was 0.8500. Furthermore, confirmation runs at a galactose:glucose ratio of 0.45 and Δ*OD*_600_ = 0.1 achieved a transferase activity of 52 U mL^−1^ (confidence interval = 35–62 U mL^−1^).

#### Increasing the Concentration of Carbon Sources

To increase transferase activity even further and suppress native invertase, we increased the glucose and galactose concentrations to 60 and 27 g L^−1^, respectively. We then monitored the Δ*OD*_600_, DO concentration, stirrer speed, and oxygen partial pressure in the gas supply over time in the MiniBio system.

**Figure 3:**
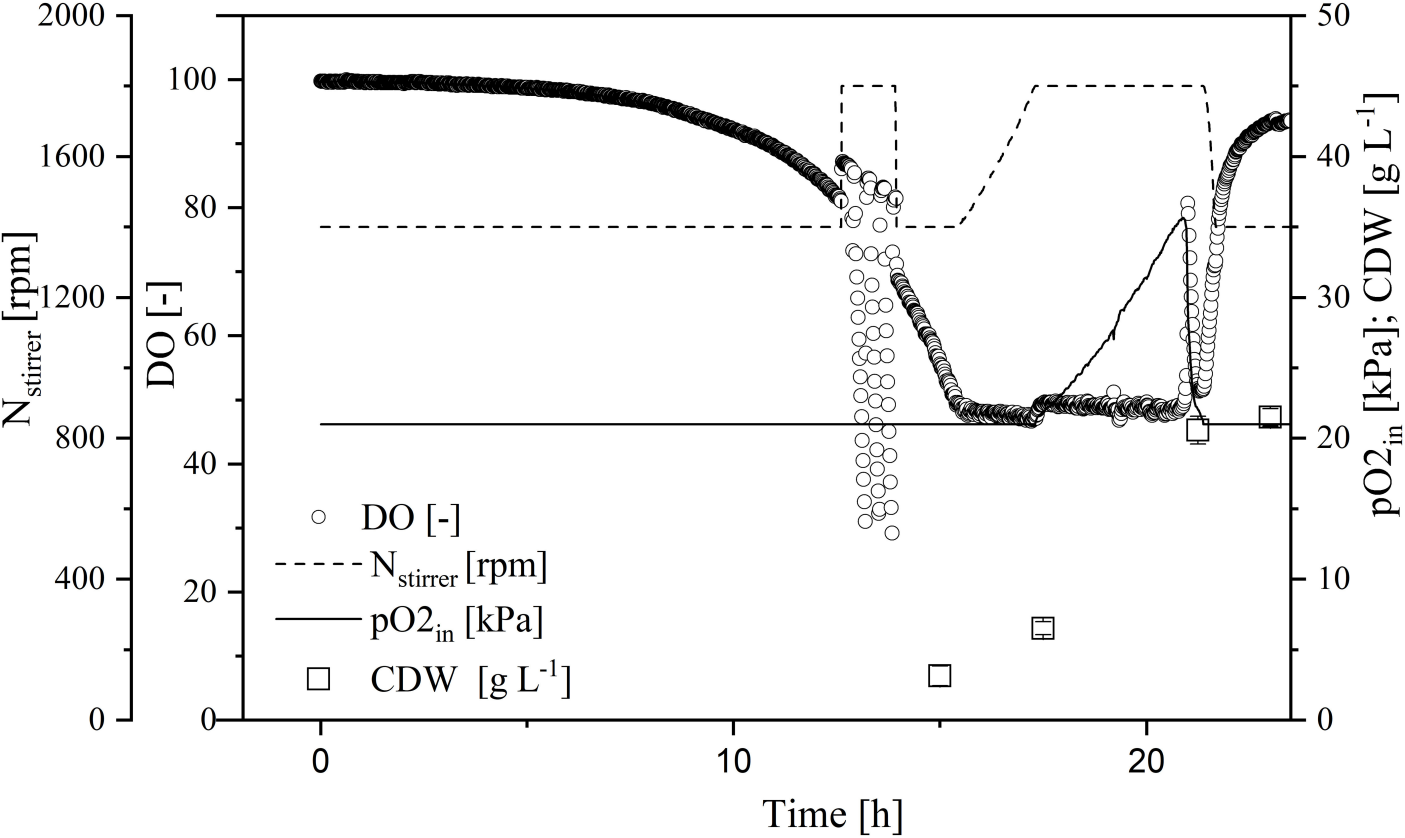
Online measurement of dissolved oxygen (DO) concentration, agitation, oxygen partial pressure in supply air, and cell dry weight (CDW) in the MiniBio system with standard medium and an adjusted inducer concentration.

**Figure 4:**
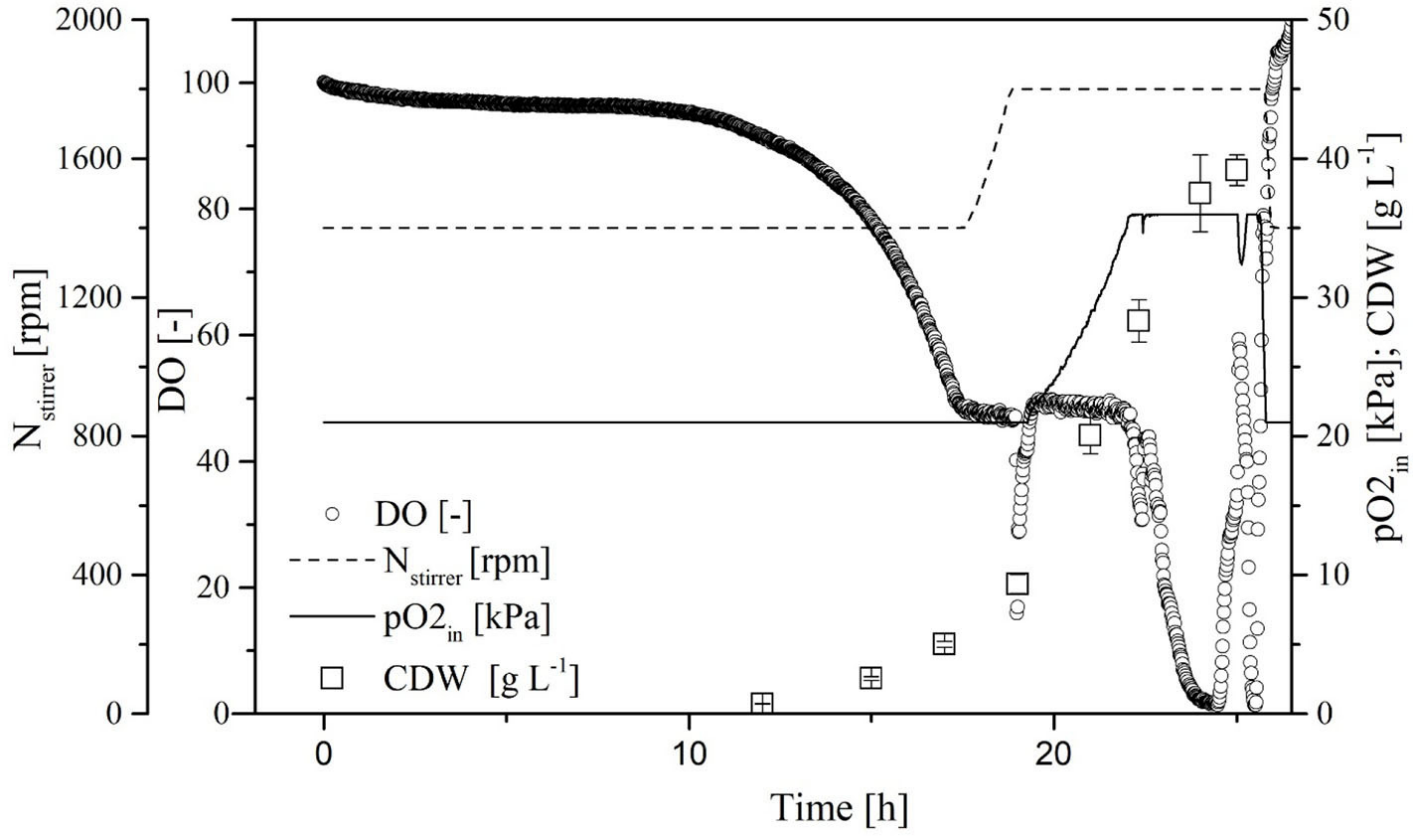
Online measurement of dissolved oxygen (DO) concentration, agitation, oxygen partial pressure in supply air, and cell dry weight (CDW) in the MiniBio system with enriched medium (higher concentration of carbon source) and adjusted inducer concentration.

A comparison of standard medium containing 30 g L^−1^ glucose (Figure 3) and the enriched medium with double the glucose concentration plus 27 g L^−1^ galactose (Figure 4) revealed an increase in CDW from 21 ± 1 to 39 ± 1 g L^−1^. Furthermore, the oxygen demand of the enriched culture was higher, so the introduced oxygen was insufficient to maintain the DO at 50% once the CDW reached ~18 g L^−1^ (Figure 4). The biomass yield decreased from 0.48 ± 0.2 to 0.45 ± 0.1 with no significant change in the growth rate. The transferase activity increased from 52 ± 5 U mL^−1^ in the standard medium to 74 ± 4 U mL^−1^ in the enriched medium, but this was not proportional to the increase in biomass, hence the specific activity decreased from 2.6 U to 1.9 U mg L^−1^. The transferase ratio increased slightly from 0.80 to 0.82.

#### Scale-Up Calculations

Scale transfer was based on geometric similarity as well as identical OTR values at the beginning of the fermentation and at the end of the oxygen control cascade. Using Equation (1), the working volume in the Labfos3 system was calculated as 3.43 L. This allowed us to calculate the maximum oxygen solubility at 30°C and oxygen partial pressures at the beginning (pO_2,t0_ = 0.21) and end (pO_2,end_ = 0.394), taking into account the main constituents of the fermentation medium. Using Equation (6) and the ionic strength and salting out coefficients of the main components, the total term for the adjusted FM22 medium (60 g L^−1^ glucose and 27 g L^−1^ galactose) can be calculated using Equation (11):

(11) $$\begin{array}{l} \log\left(\frac{\alpha_{0}}{\alpha }\right)=\sum_{\text{i}}{\text{H}}_{\text{i}}I_{i}=0.087079 \end{array}$$

Furthermore, the Bunsen coefficient α_0_ is required for pure water at 30°C, and is calculated using Equation (7) (α_0_ = 0.026354). The oxygen solubility is then calculated using Equation (12), taking into account the atmospheric pressure, the oxygen partial pressure, and the molecular weight of oxygen (32 g mol^−1^):

(12) $$\begin{array}{l} {c^{*}}_{L,1}\;=\;\frac{21.23\text{ kPa }\times \;\alpha }{22.41\;\frac{L}{\text{mol}}\;\times \;101.3\text{ kPa}}\;\times \;32\;\frac{\text{g}}{\text{mol}}=\;6.454\text{ mg }{\text{L}}^{-1} \end{array}$$

At the end of the cascade for oxygen control in the MiniBio system, the oxygen partial pressure is pO_2,end_ = 0.394, which changes the maximum oxygen solubility as shown in Equation (13):

(13) $$\begin{array}{l} {c^{*}}_{L,2}\;=\;\frac{39.9\text{ kPa }\times \;\alpha }{22.41\;\frac{L}{\text{mol}}\;\times \;101.3\text{ kPa}}\times \;32\;\frac{\text{g}}{\text{mol}}=\;12.26\text{ mg }{\text{L}}^{-1} \end{array}$$

This yields the following OTR values assuming a constant DO of 50% and the K_L_a values from Table 1: *OTR*_1_ = 0.33 g L^−1^ h^−1^ and *OTR*_2_ = 1.15 g L^−1^ h^−1^. An aeration rate of 1 vvm and a stirrer speed of 1,200 rpm produces a maximum pO_2_ of 40.1 so that, at a DO of 50%, OTR_2_ = 1.15 g L^−1^ h^−1^. The starting conditions at the same OTR_1_ were 800 rpm and 1 vvm. The results of the experimentally determined oxygen solubility when gassed with air using the H_2_O_2_ method ($c_{L}^{*}\;=$ 6.34 mg_O2_ L^−1^) and during a selected fermentation ($c_{L}^{*}\;=$ 6.23 ± 0.63 mg_O2_ L^−1^) suggested that the theoretical calculation can be used for process development (see Supplementary Material).

#### Characterization of the Transferred Process

**Figure 5:**
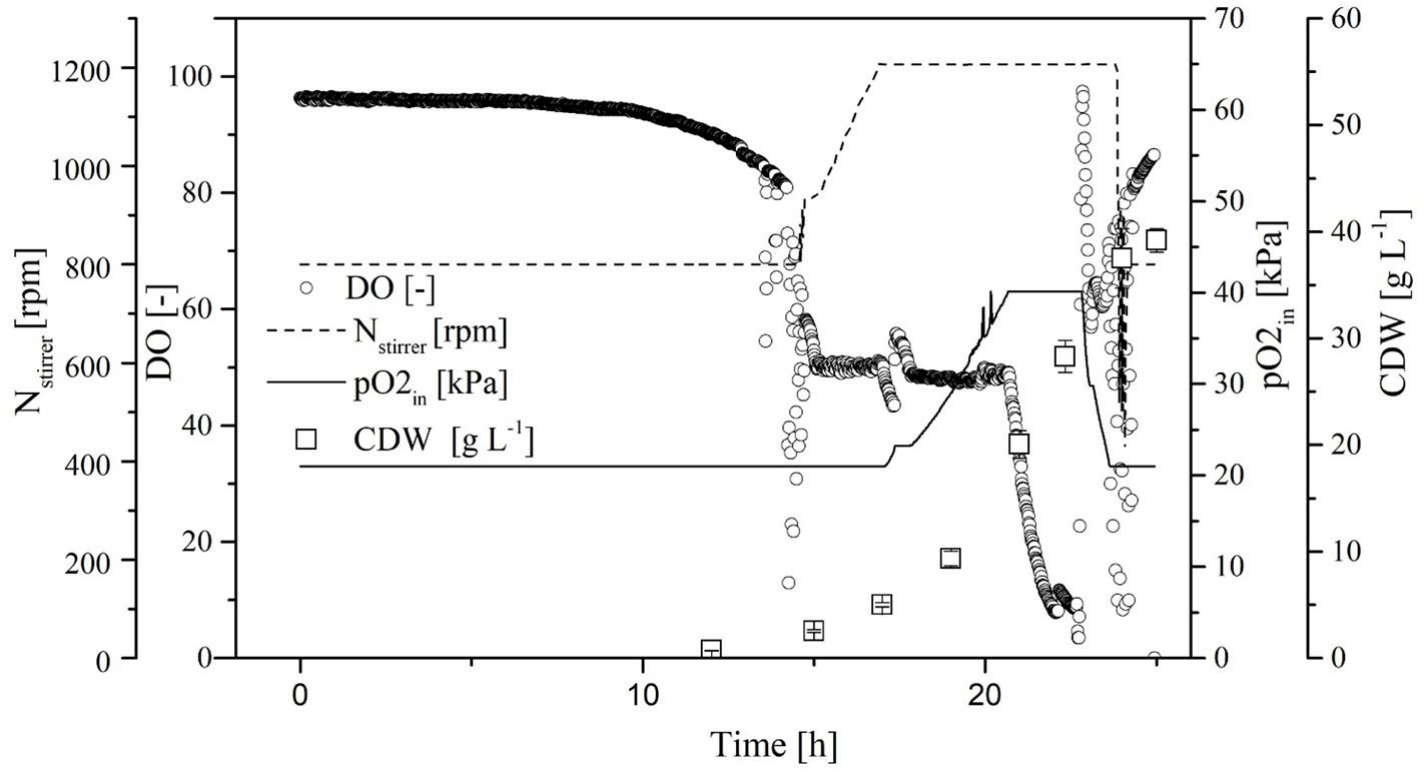
Course of the online dissolved oxygen (DO) concentration, agitation, oxygen partial pressure in the supply air, and cell dry weight (CDW) in a Labfors3 reactor based on the OTR as a scale-up criterion.

The process transferred to the 7-L reactor was characterized to evaluate the scale transfer. For this purpose, we considered the course of the process as well as the biological parameters of the biomass, growth rate, yield coefficients, and volume activity of the heterologous FFT. Initially, the CDW increased exponentially, reaching 40.4 ± 0.4 g L^−1^ at the end of the cultivation. When the CDW reached 3 g L^−1^, the DO was at 50% so that the oxygen control cascade was activated (Figure 5). By increasing the agitation from 800 to 1,200 rpm and the pO_2_ of the supply air to 40.1%, the DO could be maintained at 50% up to a CDW of 18.7 ± 0.2 g L^−1^. The DO subsequently fell below 10%. During this time, the transferase activity remained constant at ~65 ± 4 U mL^−1^ despite a further increase in CDW. During the stationary phase under high oxygen concentrations (DO > 50%) the transferase activity increased to 85 ± 3 U mL^−1^.

#### Evaluation of the Scale Transfer

**Figure 6:**
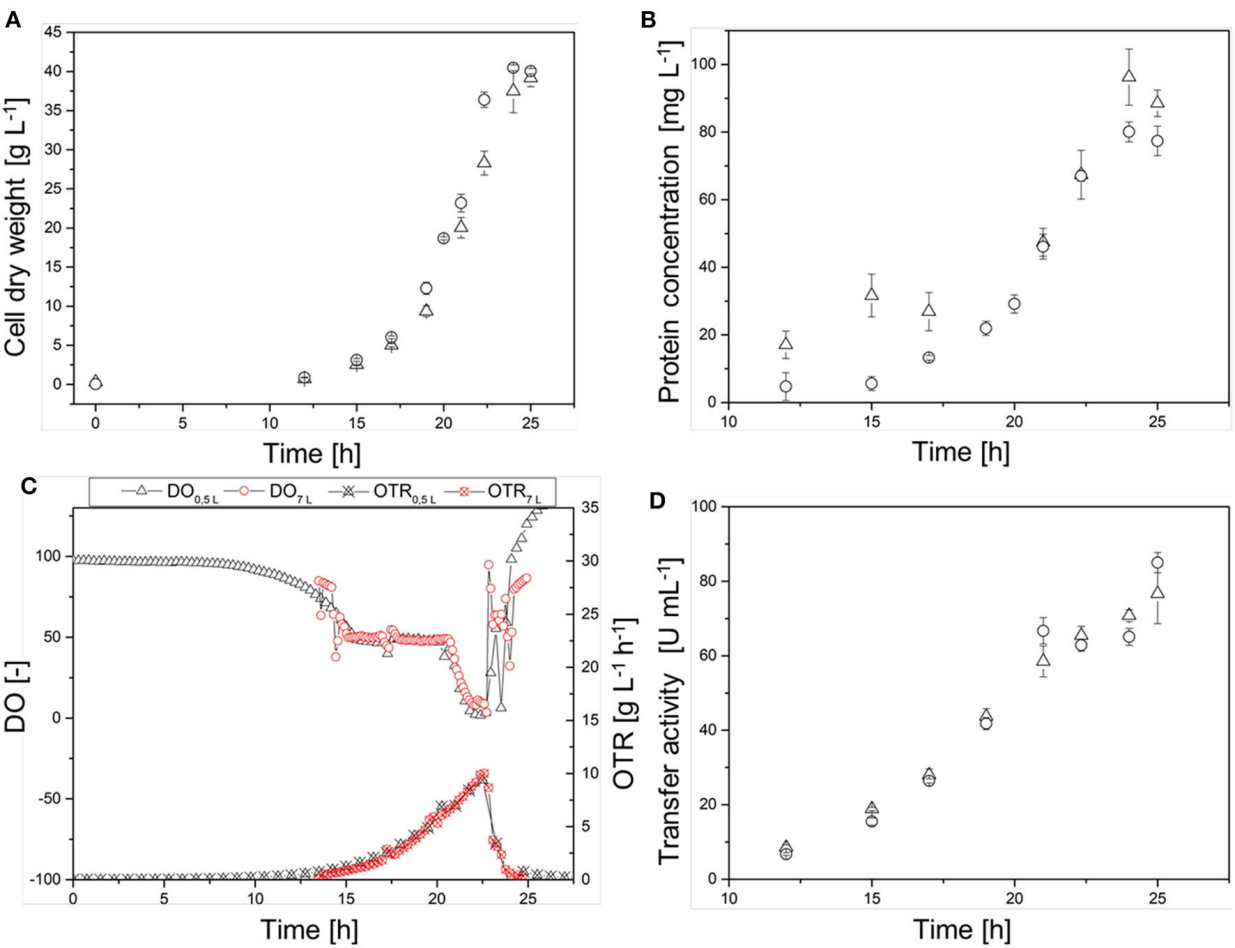
Comparison of the production process in the 0.5-L reactor (Δ) and in the 7-L reactor (◦) on the basis of **(A)** the cell dry weight (CDW), **(B)** the protein concentration, **(C)** the dissolved oxygen (DO) concentration and online oxygen transfer rate (OTR), and **(D)** of the transferase activity. Different lag phases were adjusted in the figures.

The scale transfer was verified by monitoring the temporal courses of CDW, protein concentration, and transferase activity in the cell-free crude enzyme solution, as well as the DO concentration and online OTR in both reactor systems (Figure 6). The CDW curves (Figure 6A) showed a similar course, although the 7-L Labfors3 system reached the maximum CDW (40.0 ± 0.3 g L^−1^) earlier than the 0.5-L reactor (39 ± 1 g L^−1^). The growth rates were μ = 0.39 ± 0.02 and 0.37 ± 0.01 for the Labfors3 and MiniBio systems, respectively. Initially, the protein concentration (Figure 6B) increased more sharply in the 0.5-L reactor (31 mg L^−1^ after 15 h) than the 7-L reactor (6 mg L^−1^ after 15 h), but from 17 h after inoculation both protein concentrations followed the corresponding CDW growth curves. The maximum protein concentration was 96 ± 8 mg L^−1^ in the 0.5-L system and 80 ± 3 mg L^−1^ in the 7-L system.

The time courses of the DO concentration in the fermentation broth and the online OTR were very similar (Figure 6C). After ~14 h, both reactor systems reached DO = 50% and the cascade of oxygen control and thus the OTR started to increase. After ~20.5 h, the DO fell below 50% in both reactor systems, and ultimately it dropped below 5% in the 0.5-L reactor and below 10% in the 7-L reactor. The maximum OTR was reached after 22.5 h in both systems. This was 10 g L^−1^ h^−1^ for the 0.5-L reactor and 11 g L^−1^ h^−1^ for the 7-L reactor. As the DO concentration increased, the OTR decreased due to the oxygen control cascade. The transferase activity in the cell-free crude enzyme solution showed an almost linear time course, with stagnation after 21 h at ~65 U mL^−1^ (Figure 6D). At the end of the fermentation, the transferase activity was 76 ± 8 U mL^−1^ in the 0.5-L reactor and 85 ± 3 U mL^−1^ in the 7-L reactor.

#### Characterization of the Transferred Process With Increased OTR

**Figure 7:**
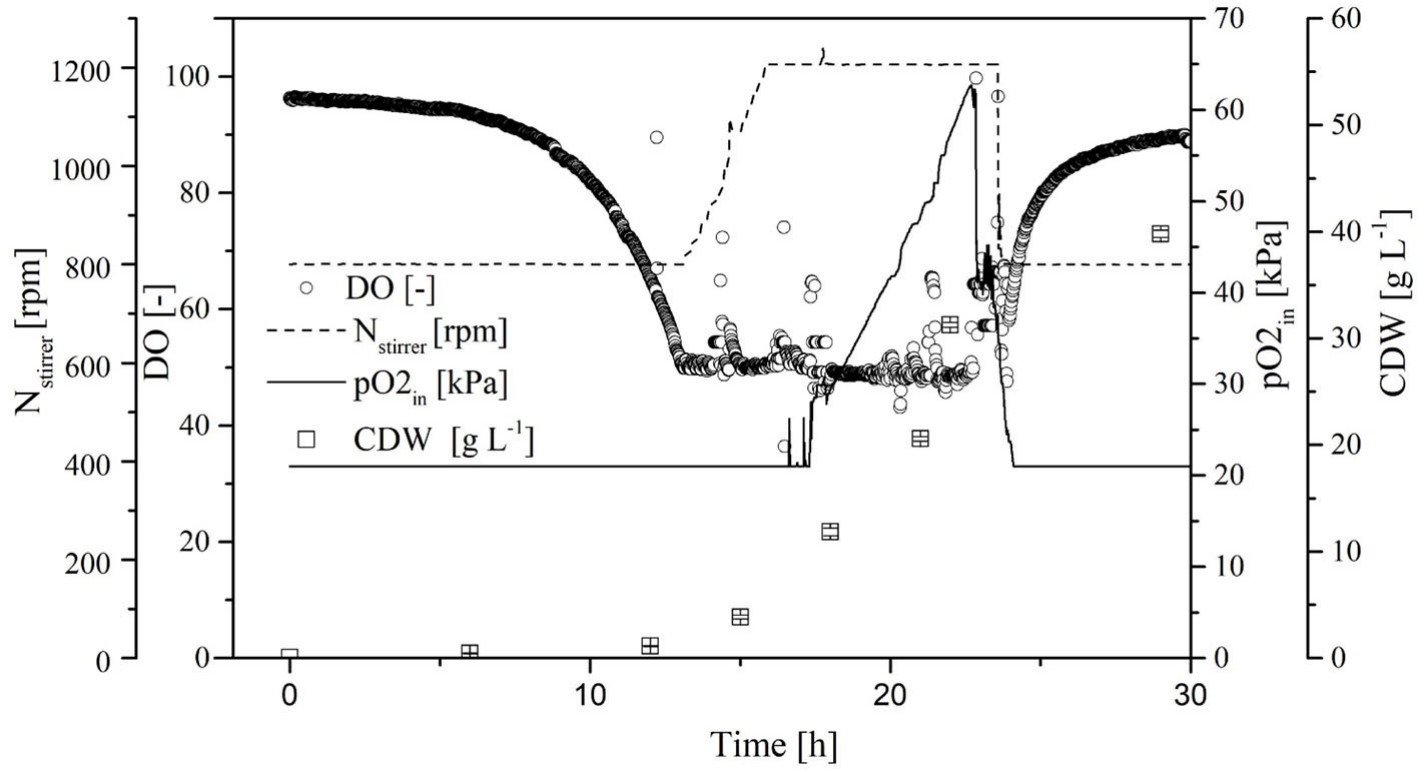
Time course of the online measured dissolved oxygen (DO) concentration, agitation, oxygen partial pressure in the supply air (pO_2_,_in_), and cell dry weight (CDW) in the Labfors3 system with increased oxygen transfer rate (OTR).

Because the specific activity is lower in the absence of oxygen, the OTR was increased to DO = 50% for stabilization. By reducing the aeration rate to 0.5 vvm and increasing the pO_2_, we were able to reduce the amount of pure oxygen added to the supply air. Initially, the CDW and transferase activity increased exponentially, and reached values of 38.5 ± 0.6 g L^−1^ and 94.0 ± 2.1 U mL^−1^, respectively, at the end of the cultivation. As soon as the DO dropped to 50%, it was kept constant by increasing the agitation rate to 1,200 rpm and increasing the pO_2_ in the supply air to 60%. When the transferase activity reached 65 U mL^−1^, further increases no longer correlated with the CDW (Figure 7).

#### Generation of the *K. lactis* GG799 *ΔInv* Mutant

The Δ*Inv* deletion was confirmed by sequencing PCR products spanning the *Inv* locus. The Δ*Inv* mutant was cultivated in the 7-L reactor using the process with the increased OTR, achieving a CDW of 40.7 ± 0.5 g L^−1^ and a transferase activity of 88 ± 7.1 U mL^−1^.

**Figure 8:**
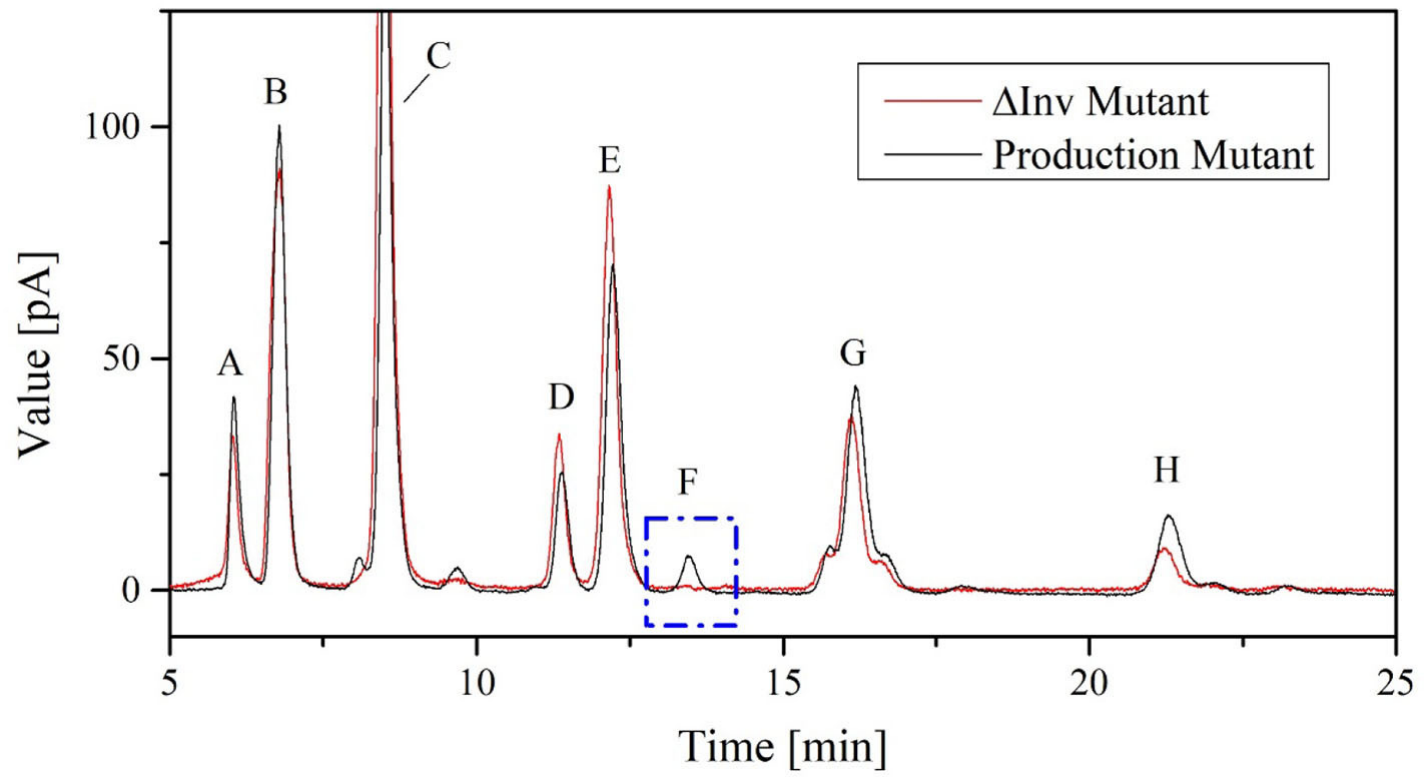
Chromatograms showing FOS catalytic products in 100 μL cell-free crude enzyme solution of *K. lactis* GG799 and GG799Δ*Inv* with 600 g L^−1^ sucrose solution for 2 h at 70°C. The peaks are annotated as follows: A = fructose, B = glucose, C = sucrose, D = neokestose, E = 1-kestose, F = 6-kestose, G = nystose, and H = 1^F^-fructofranosylnystose.

The *K. lactis* invertase forms 6-kestose during FOS biosynthesis and is responsible for increased hydrolysis activity in the crude enzyme solution of the production strain (Burghardt et al., 2019a). No 6-kestose was detected in the fermentation broth of the Δ*Inv* mutant, which provided further evidence for the successful deletion of the *Inv* locus (Figure 8).

#### Comparison of *K. lactis* GG799 and GG799*ΔInv* in Batch and Fed-Batch Processes

**Figure 9:**
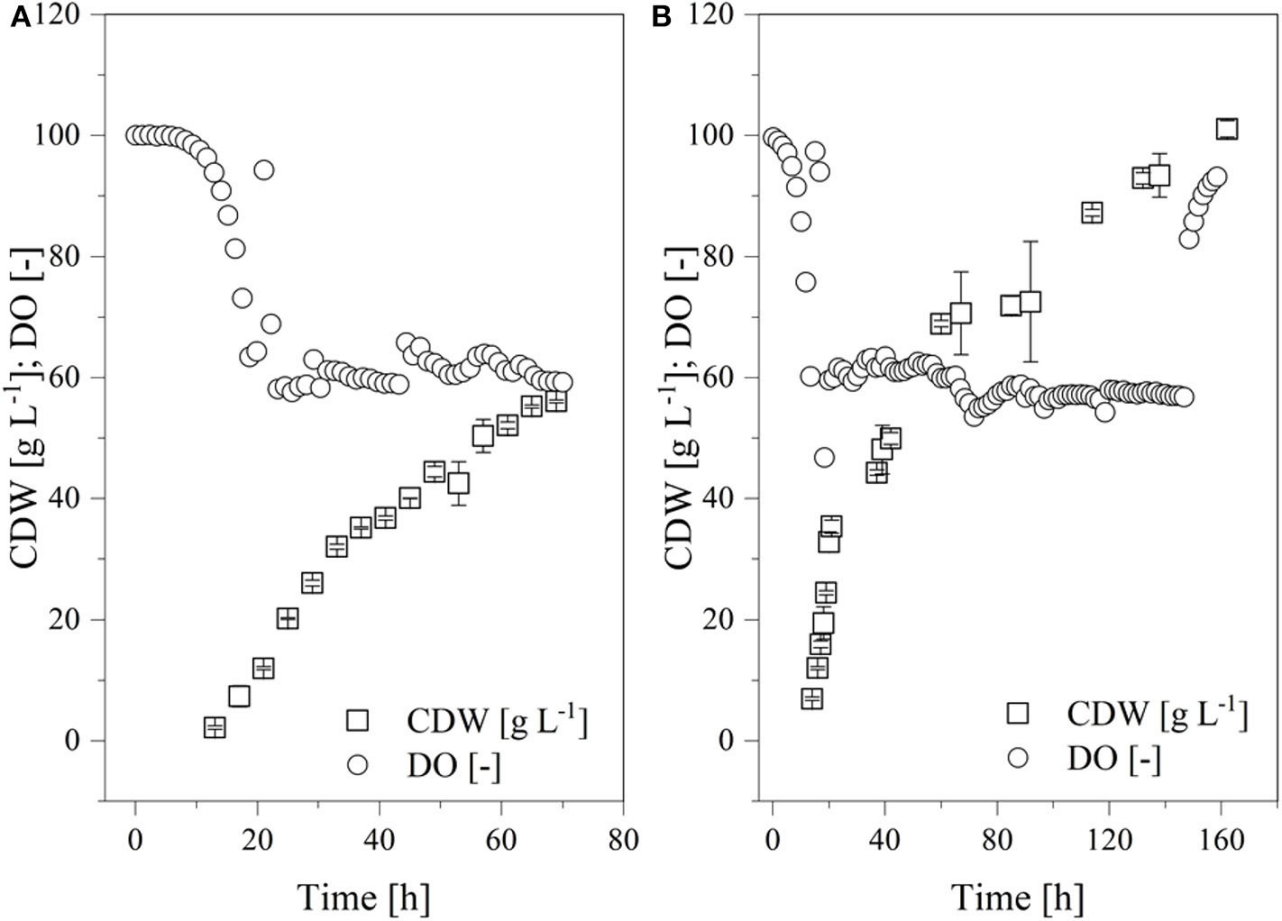
Time course of the online measured dissolved oxygen (DO) concentration and cell dry weight (CDW) in a fed-batch process using the Labfors3 system. **(A)** Production strain *K. lactis* GG799 and **(B)** mutant strain *K. lactis* GG799Δ*Inv*.

The results thus far indicated that a continuous supply of oxygen can increase the transferase activity. For this reason, fed-batch fermentations were established at the 7-L scale for *K. lactis* GG799 and GG799Δ*Inv* at a continuous DO of ~60% for comparison (Figure 9). Both processes began with an initial batch mode at 3.43 L. The adapted FM22 medium was used, but initially only 10 g L^−1^ glucose and 4.5 g L^−1^ galactose were provided to prevent the DO falling below 50% during the batch phase. When the carbon source was depleted, the DO increased and the fed-batch phase began. A PID controller was used to adjust the feed rate so that the DO remained at ~60%. Given the increased oxygen demand of fermentations at μ_max_, the growth rate in these experiments was reduced by aeration using air without an additional oxygen supply. We provided filter-sterilized 300 g L^−1^ glucose and 135 g L^−1^ galactose as feed solutions.

The batch phase of both strains ended after ~20 h and feed control was started. The *K. lactis* GG799 production strain reached a CDW of 57.8 ± 4.2 after 70 h and the GG799Δ*Inv* mutant reached a CDW of 101.1 ± 1.37 after 162 h. For better comparability, a sample was also taken during the fermentation of the GG799Δ*Inv* mutant at a CDW of 57.8 g L^−1^ and transferase activities at all three points were determined. The activity after 20 min was 414.7 ± 11.7 U mL^−1^ for the production strain, and 450.1 ± 23.6 U mL^−1^ (CDW = 57.7) or 557.0 ± 36.2 U mL^−1^ (CDW = 101 g L^−1^) for the Δ*Inv* mutant (Table 3).

**Table 3:** Comparison of transferase activities in batch and fed-batch mode after 20 min (*n* = 3).

**Production mode [-]**	**Strain [-]**	**Transferase activity [U mL^**−1**^]**
Batch	GG799	333.7 ± 28.6
Batch	GG799*ΔInv*	341.6 ± 20.1
Fed-batch (CDW = 58 g L^−1^)	GG799	414.7 ± 11.7
Fed-batch (CDW = 58 g L^−1^)	GG799*ΔInv*	450.1 ± 23.6
Fed-batch (CDW = 101 g L^−1^)	GG799*ΔInv*	557.0 ± 36.2

**Figure 10:**
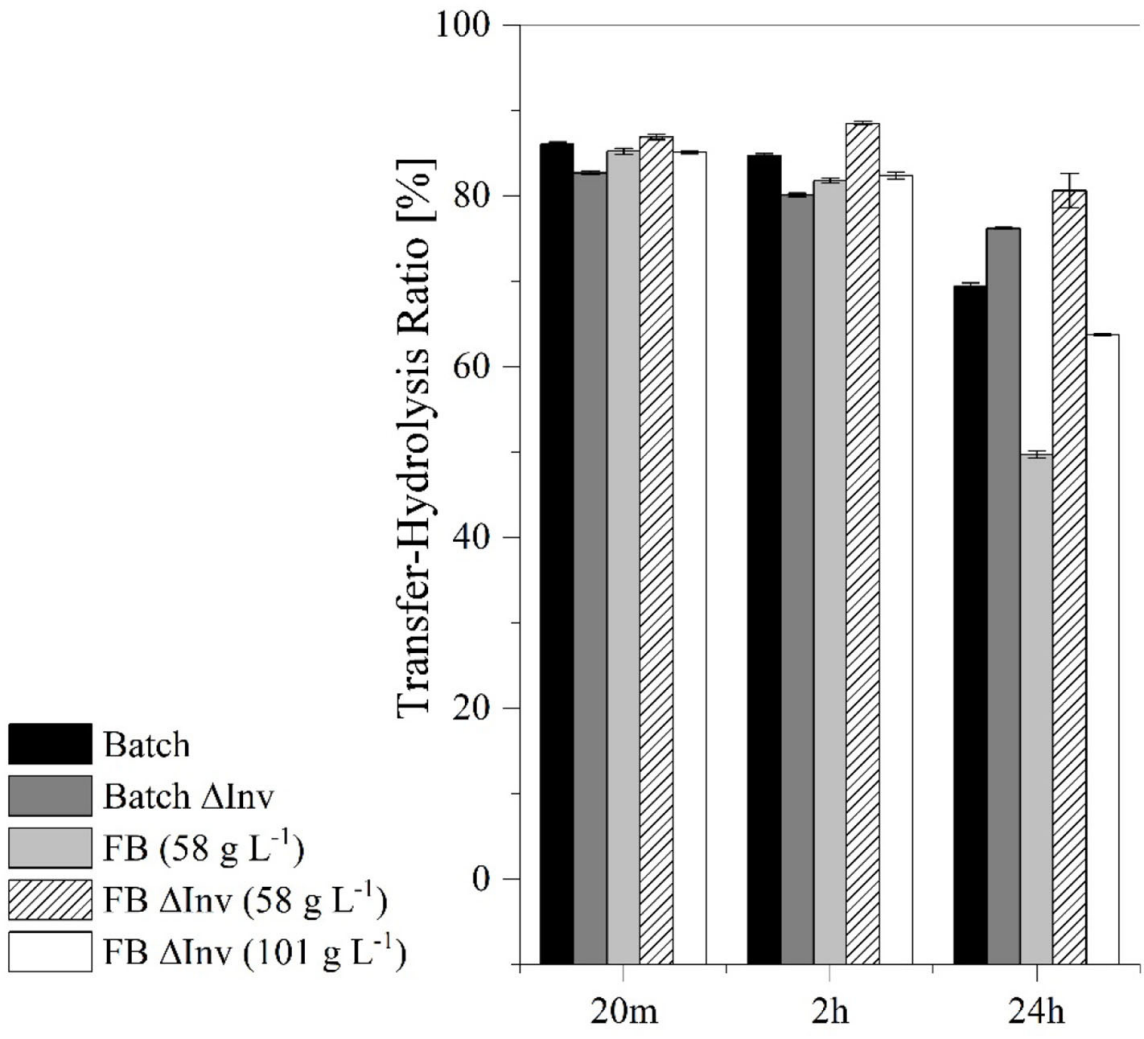
Comparison of the transferase-hydrolysis ratio in catalytic reactions lasting 20 m, 2 h and 24 h using batch and fed-batch (FB) processes with the original production strain and the Δ*Inv* mutant. The error bars represent the standard deviation of triplicates.

The resulting activities after 20 m in fed-batch mode were also compared to the corresponding activities in batch mode with the increased OTR (Table 3). The fed-batch fermentation with the GG799Δ*Inv* mutant achieved 66.9% higher transferase activity than the batch fermentation with the original production strain. We found a significant influence (*p* = 0.001) of invertase deletion considering the transferase-hydrolysis ratios after 24 h of catalysis. For this purpose, we compared the production (49.7 ± 0.4%) and GG799ΔInv strain in fed-batch mode (Figure 10).

These results suggested that higher transferase activity could achieved by fermentation under aerobic conditions using the GG799Δ*Inv* mutant (Table 3). The ratio between transferase and hydrolysis activities was determined after reactions lasting 20 min, 2 h and 24 h (Figure 10). We found that the hydrolysis reaction was more prevalent after 24 h, particularly for the original production strain in the fed-batch process, which resulted in the lowest transferase-hydrolysis ratio we observed (49.7 ± 0.4%).

## Discussion

We investigated process conditions that favor transferase over invertase activity in the yeast strain *Kluyveromyces lactis* GG799 during a scaled-up fermentation process. The carbon source was selected to maximize both the CDW and transferase activity. This was achieved with a mixture of glucose, which represses native invertase activity (Bandyopadhyay et al., 1967; Kuzhandaivelu et al., 1992; Georis et al., 1999) and galactose to induce the *LAC4* promoter. Galactose was a superior inducer to lactose, increasing the transferase activity from 92 ± 23 to 166 ± 11 U mL^−1^ based on a reaction time of 20 min. This may reflect the stronger activation of the promoter due to higher binding constants or lower dissociation constants for the corresponding transcriptional activator in the presence of galactose (Daber et al., 2007) and/or the higher molarity of the monosaccharide galactose given that both sugars were added with the same mass ratio and lactose is a disaccharide. Galactose can also be taken up into the cell via separate transporters, achieving a higher intracellular concentration than lactose (Breunig et al., 2000). Having selected galactose as the inducer, we found that productivity was reduced at lower galactose:glucose ratios or when induction was delayed. By increasing the induction ratio from 0.25 to 0.45, the transferase activity increased from 40 to 52 U mL^−1^. The higher sugar concentration at greater induction ratios provides more energy and thus increases transferase expression. However, the transferase activity was higher than anticipated from the increase in carbon concentration alone. Even very low permanent galactose concentrations of 0.5 g L^−1^ can activate the *LAC4* promoter (Panuwatsuk and Da Silva, 2003). For this reason, a constant galactose concentration could be achieved by feeding to save galactose (Panuwatsuk and Da Silva, 2003). Having established the optimum induction ratio, we doubled the concentration of both carbon sources to 60 g L^−1^ glucose and 27 g L^−1^ galactose. This increased transferase activity by 50% to 78 U mL^−1^ and the CDW increased by 86%. However, the DO concentration of 50% could not be maintained due to the increased oxygen demand, potentially explaining why the biomass yield coefficient declined from 0.48 to 0.44 in Crabtree-negative yeast (González Siso et al., 1996). Furthermore, the uptake of galactose can be greatly reduced under anaerobic conditions, such that the intracellular concentration is insufficient for induction (Goffrini et al., 2002).

The reactor systems were characterized so that calculations could be based on correct assumptions or in the working area of empirical equations. The design of the reactors was based on the limits of the oxygen input so that the target value of the maximum CDW could be determined at a DO > 50%, while gassing with air and during growth at μ_max_. The CDW was 3–20g L^−1^ for the MiniBios system and 3–26 g L^−1^ for the Labfors3 system for coalescent and non-coalescent media. Although saline aqueous solutions generally tend to be non-coalescent systems, coalescence is strongly promoted by antifoaming agents (Morão et al., 1999). Because coalescence has a significant influence on k_L_a (Table 1), the true value does not represent either of the two extreme cases (Figures 5–7). Although the error is assumed to be <6% (Van't Riet, 1979), a significant reduction in k_L_a was observed by dynamic outgassing if the reaction time of the probe was ignored (Tribe et al., 1995). However, if similar reaction times of the DO probes are assumed, the correction can be omitted (Tribe et al., 1995). This assumption was confirmed in our experiments by the similar course of the OTR in both reactor systems (Figure 6C). By outgassing with microorganisms, the critical oxygen concentration at which metabolism is no longer completely based on respiration was set at DO = 25% (1.62 mg L^−1^). This value is 2-fold higher than the literature values of c_O2, crit_ reported for the related yeast *Kluyveromyces marxianus* (Cho and Wang, 1990) and more than 10-fold higher than the values reported for *Escherichia coli* (Chen et al., 1985). Because the critical oxygen concentration refers to the oxygen consumption or growth rate, it is only a first indication for the design of the bioreactor. Furthermore, the influence of oxygen on product formation or CDW must be investigated. For *K. lactis*, a DO of 40–60% in chemically defined medium was previously shown to achieve the highest CDW and product yields (Blondeau et al., 1994).

The scale transfer was based on the OTR as the critical and limiting parameter. The working volume of 3.43 L was calculated using geometric similarity. Subsequently, the experimental determination of K_L_a using a response surface model, and the calculated solubility of the medium based on the main components of $c_{L}^{*}\;$ = 6.454 mg L^−1^, allowed us to determine the settings for an identical OTR. An OTR of 1.15 g L^−1^ h^−1^ was achieved at 1,200 rpm, 1 vvm and pO_2_ = 40.1. The evaluation of the scale transfer was carried out using the time courses of CDW, protein concentration, transferase activity, DO, and OTR.

In the Labfors3 system, the culture reached stationary phase shortly before the culture in the MiniBio system, but this was probably due to fluctuating inoculation densities and lag phases given that the growth rates did not differ significantly. Both systems reached a similar CDW. Both courses of protein concentration followed the CDW and reached values of 96 ± 8 mg L^−1^ in the 0.5-L reactor and 80 ± 3 mg L^−1^ in the 7-L reactor. The transferase activity was initially very similar in both systems, but reached 85 ± 3 U mL^−1^ in the 7-L reactor and 76 ± 8 U mL^−1^ in the 0.5-L reactor by the end of the fermentation. These differences in CDW and transferase activity may reflect the slightly lower oxygen supply in the MiniBio system. The DO in the MiniBio system fell below 5% between 22 and 24 h, but remained at 5–10% in the Labfors3 system over the same period (Figure 6C). Given that anaerobic metabolism produces less energy, this can have a negative effect on heterologous protein expression and biomass formation. These differences in the minimum DO may be caused by inaccurate K_L_a measurements. This was shown by a slightly higher OTR of the Labfors3 system (10.1 g L^−1^ h^−1^) compared to the MiniBio system (9.5 g L^−1^ h^−1^). The similarity between the reactor systems is granted, ignoring time differences due to fluctuations in the lag phase, so that the scale increase was considered successful.

By increasing the OTR in the Labfors3 system, the DO remained at or above 50% at all times so that oxygen had no limiting effect, and the transferase activity increased from 85 ± 3 to 94 ± 2 U mL^−1^. There may be a correlation with the stagnation of transferase activity in the cell-free crude enzyme solution during oxygen deficiency (Figure 6). However, the galactose was consumed well before the glucose, so that strong growth still took place without sufficient galactose induction. Due to the accumulation of metabolites such as ethanol or glycerol in the partially anaerobic metabolism (Merico et al., 2009), the energy yield is lower under conditions of sufficient galactose induction. The aerobic metabolism of these metabolites could then, without galactose as an inducer, inhibit heterologous protein expression. Without oxygen as a limiting substrate, doubling the carbon concentration approximately doubled the transferase activity. The biomass decreased by 4.5% at a continuous DO > 50%. This may be due to the kinetics of cell death and the associated decrease in CDW, given that harvesting took place only 6 h after the beginning of the stationary phase.

After catalysis for 24 h, all cell-free crude enzyme solutions showed a reduced transferase/hydrolysis ratio, whereas the lowest ratio with 49.7 ± 0.4% was observed after the fed-batch fermentation with the production strain (Figure 10). Fed-batch fermentation under conditions of carbon source limitation therefore provides insufficient quantities of glucose during fermentation to repress the production of native invertase (Kuzhandaivelu et al., 1992). Accordingly, the equivalent process using the Δ*Inv* mutant (at the same CDW) achieved a transferase/hydrolysis ratio of 76.2 ± 0.4%, which is 55.1% higher than the production strain. But this represents a decrease of 26.5% when both CDWs are taken into account, suggesting that both enzymes in the crude enzyme solution show transferase and hydrolysis activity. Because higher glucose concentrations can inhibit the transferase activity of the enzymes, systems such as enzyme membrane reactors may be needed for further process development so that the residence time of the enzymes can be adjusted to further optimize the transferase/hydrolysis ratio (Erdos et al., 2017; Burghardt et al., 2019b; Fan et al., 2020).

## Conclusion

We improved the heterologous expression of *A. terreus* FFT in *K. lactis* GG799 during batch-mode fermentations, increasing the transferase activity and providing the basis for us to scale up the process from 0.5 to 7 L. We optimized the carbon source, using glucose as the principal carbon source and inhibitor of invertase, and galactose as the secondary carbon source and inducer of FFT expression given its superior performance compared to lactose. The addition of twice the carbon concentration caused oxygen limitation, so that transferase activity increased by only ~50%. The critical OTR parameter was therefore used for the scale-up, resulting in a geometrically similar bioreactor with an identical OTR. This achieved similar results in terms of CDW, protein concentration and transferase activity, as well as DO and online OTR. The OTR was then increased to maintain the DO > 50%, resulting in a transferase activity of 94 U mL^−1^ (169% higher than the standard process with 30 g L^−1^ glucose and 7.5 g L^−1^ galactose). Deleting the native invertase gene led to a further 66.9% increase in transferase activity in fed-batch cultivations. However, our results also suggested that FFT possesses intrinsic invertase activity, and reaction systems for large-scale FOS production should therefore optimize the residence time of the enzyme to favor transferase activity.

## Data Availability Statement

The datasets presented in this study can be found in online repositories. The names of the repository/repositories and accession number(s) can be found in the article/Supplementary Material.

## Author Contributions

JB designed and performed the experiments and wrote the paper. RF helped to draft the manuscript and assisted with the experiments. MB assisted with the experiments. DE helped to design the experiments. DG and PC helped to draft and revise the manuscript and supervised the research. All authors contributed to manuscript revision, and they read and approved the submitted version.

## Conflict of Interest

The authors declare that the research was conducted in the absence of any commercial or financial relationships that could be construed as a potential conflict of interest.
