# Supplementary material for: Enhancing the Heterologous Fructosyltransferase Activity of Kluyveromyces lactis: Developing a Scaled-Up Process and Abolishing Invertase by CRISPR/Cas9 Genome Editing
Source: Front Bioeng Biotechnol. 2020 Nov 25;8:607507. doi: 10.3389/fbioe.2020.607507 (PMC7724039; doi:10.3389/fbioe.2020.607507)
Supplement: Supplementary file 1 [file Data_Sheet_1.docx]

Supplementary Material

**Enhancing the heterologous fructosyltransferase activity of *Kluyveromyces lactis*: developing a scaled-up process and abolishing invertase by CRISPR/Cas9 genome editing**

**Jan Philipp Burghardt^1,2^, Rong Fan^3^, Markus Baas^1^, Dustin Eckhardt^1^, Doreen Gerlach^3^, Peter Czermak^1,2,3^***

^1^Institute of Bioprocess Engineering and Pharmaceutical Technology, University of Applied Sciences Mittelhessen, Giessen, Germany

^2^Faculty of Biology and Chemistry, Justus Liebig University, Giessen, Germany

^3^Department of Bioresources of Fraunhofer Institute for Molecular Biology and Applied Ecology IME, Giessen, Germany

*** Correspondence:**Prof. Dr.-Ing. Peter Czermak
peter.czermak@lse.thm.de

# Measuring the K_L_a and oxygen saturation concentration

To determine the K_L_a in the Infors3 system, aeration was switched off and the pO_2_ curve was recorded (Supplementary Figure 1A). The logarithm of the difference between the starting pO_2_ value and the pO_2_ at time t (pO_2,(t)_) of gassing out was calculated and plotted against the time (Supplementary Figure 1B). The slope corresponds to the K_L_a after extrapolation to 1 min^-1^.


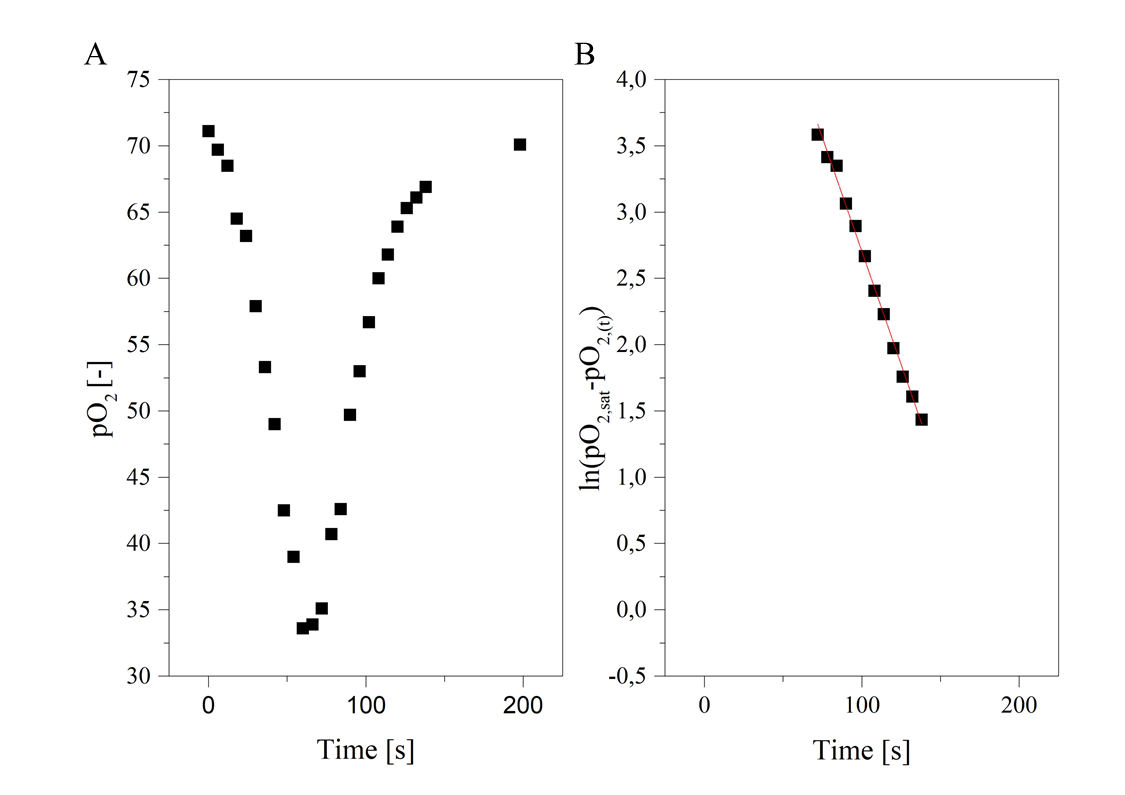


Supplementary Figure 1: Representative A pO_2_ curve and B logarithmic differences between the starting pO_2_ and pO2_(t)_ at time t of a measurement. The curves were recorded during outgassing with *K. lactis* (Δ*OD_600_* = 30–40) with an aeration rate of 0.5 vvm and an agitation rate of 1200 rpm in a working volume of 3.43 L.

The K_L_a calculations were repeated in a response surface design by investigating an aeration range of 0.5–1.5 vvm and an agitation range of 800–1200 rpm (Supplementary Figure 2). ANOVA of the K_L_a predictive model showed the model is significant (p = 0.0005) with a non-significant lack of fit (p = 0.9066).

rpm
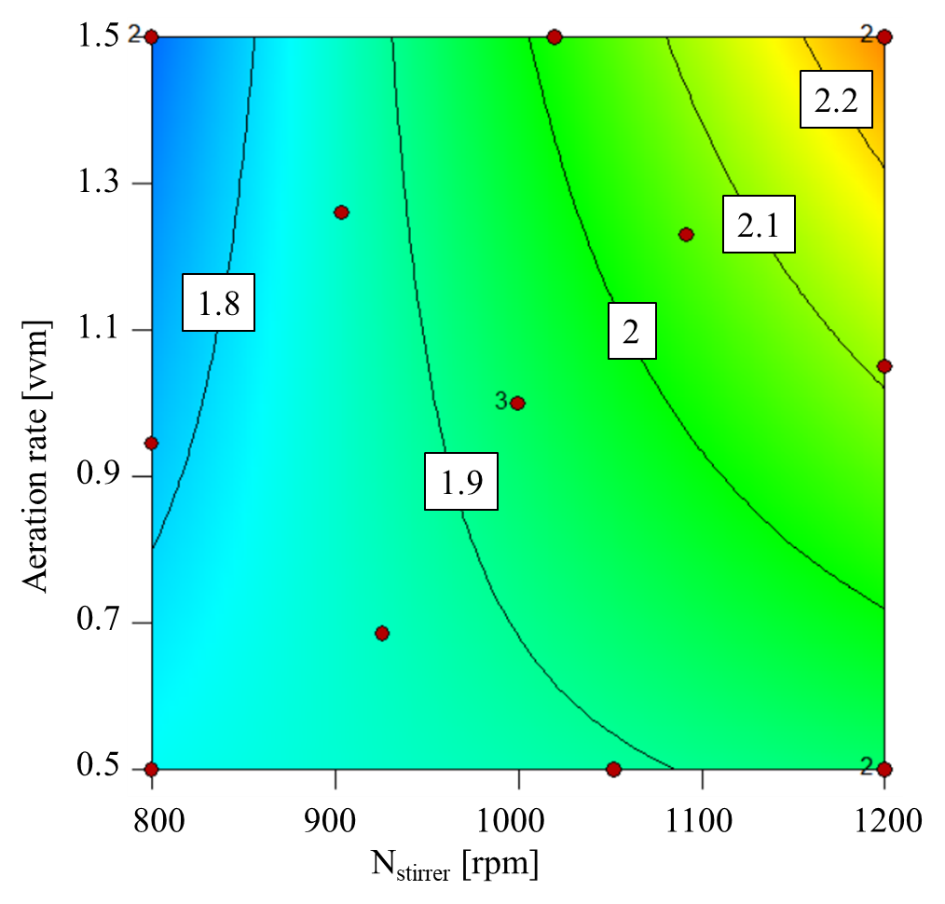


Supplementary Figure 2: Contour plot of the predictive model for K_L_a (min^-1^) with the influencing factors stirrer speed and aeration rate within the experimental limits.

# Determination of oxygen solubility in the fermentation medium: Probe response of DO to increased H_2_O_2_ volumes

The oxygen saturation was determined as described by Vendruscolo et al. (2012). The probe response after adding known amounts of H_2_O_2_ is shown in Supplementary Figure 3. The probe was calibrated to 100% by aeration with air to equilibrium. The oxygen was gassed out with nitrogen before each measurement.


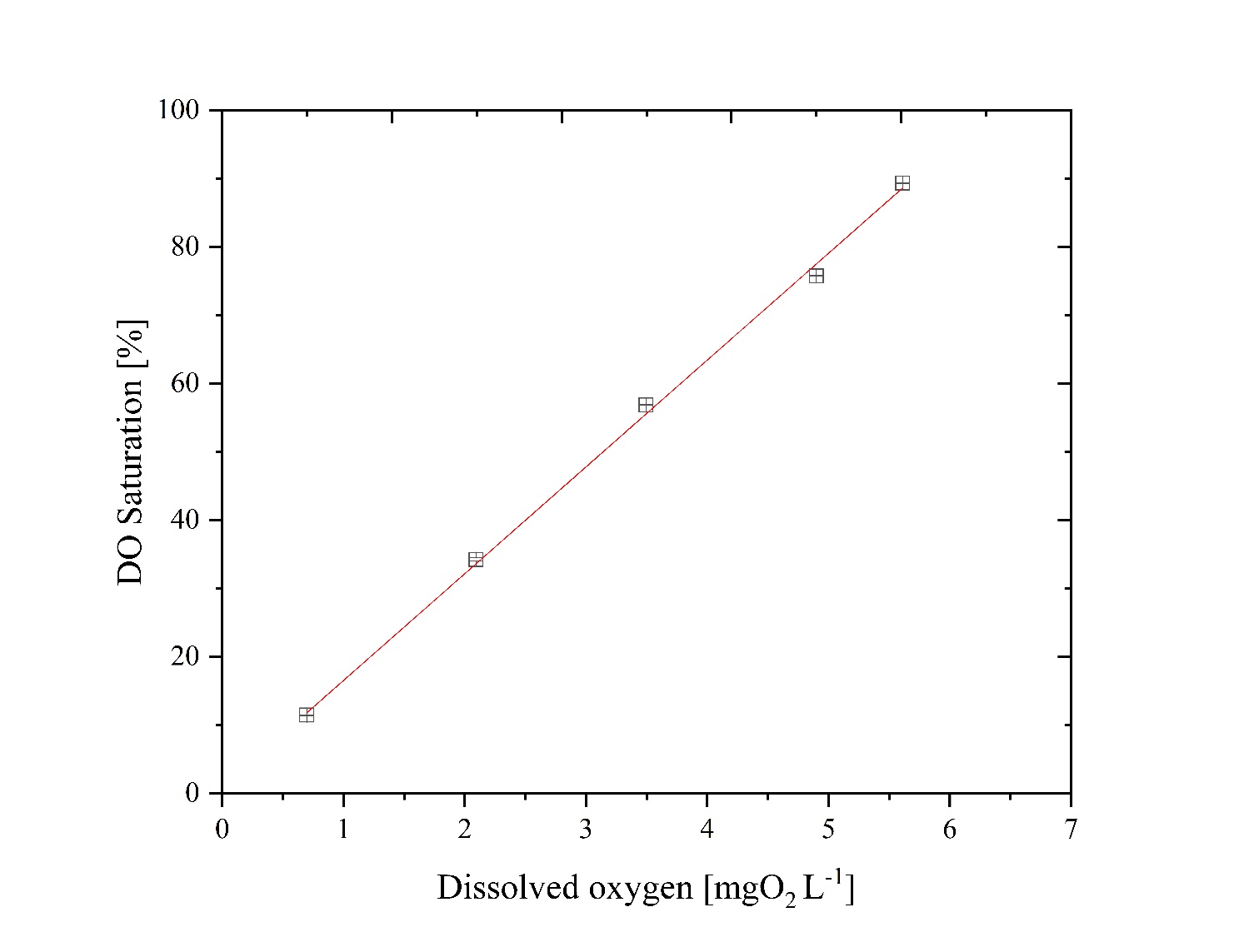


Supplementary Figure 3: Probe response of dissolved oxygen at 30 °C in cell-free fermentation medium.

The experimental data points were fitted to Supplementary Eq. 1 (R^2^ = 0.99921):

DO saturation = 0.9 + DO $\boldsymbol{\times}$ 15.63 Supplementary Equation 1

Using Supplementary Eq.1, the oxygen solubility at 100% saturation in the cell-free fermentation medium was 6.34 mg_O2_ L^-1^.

# Determination of oxygen solubility in the fermentation medium: Estimation during the cultivation of *K. lactis*

To estimate the oxygen solubility, the aeration was switched off during fermentation with a known CDW. Based on the percentage decrease of the DO, the saturation concentration of oxygen was determined assuming that the DO probe response at 100% corresponds to the saturation concentration of O_2_ in the medium when aerated with air. Shortly before recording the DO drop, the average $\bar{OTR}$ value was determined by exhaust gas analysis (Supplementary Eq.2). We assumed that $\bar{OTR}$ = $\bar{OUR}$ because, during $\bar{OTR}$ recording, the change of DO was negligible (< 2%).

$\bar{OTR}= \bar{OUR}=0.53\left[ \frac{g_{O_{2}}}{L \times h} \right]=8.83\left[ \frac{{mg}_{O_{2}}}{L \times min} \right]$ **Supplementary Equation** **2**

With the known oxygen uptake rate and CDW, the specific oxygen uptake rate $q_{O_{2}}$was determined (Supplementary Eq. 3).

$q_{O_{2}}=\frac{\bar{OUR}}{\bar{CDW}}=\frac{8.83 \left[ \frac{{mg}_{O_{2}}}{L \times min} \right]}{2.46 \left[ \frac{g_{CDW}}{L} \right]}=3.59 \left[ \frac{{mg}_{O_{2}}}{g_{CDW} \times min} \right]$ **Supplementary Equation 3**


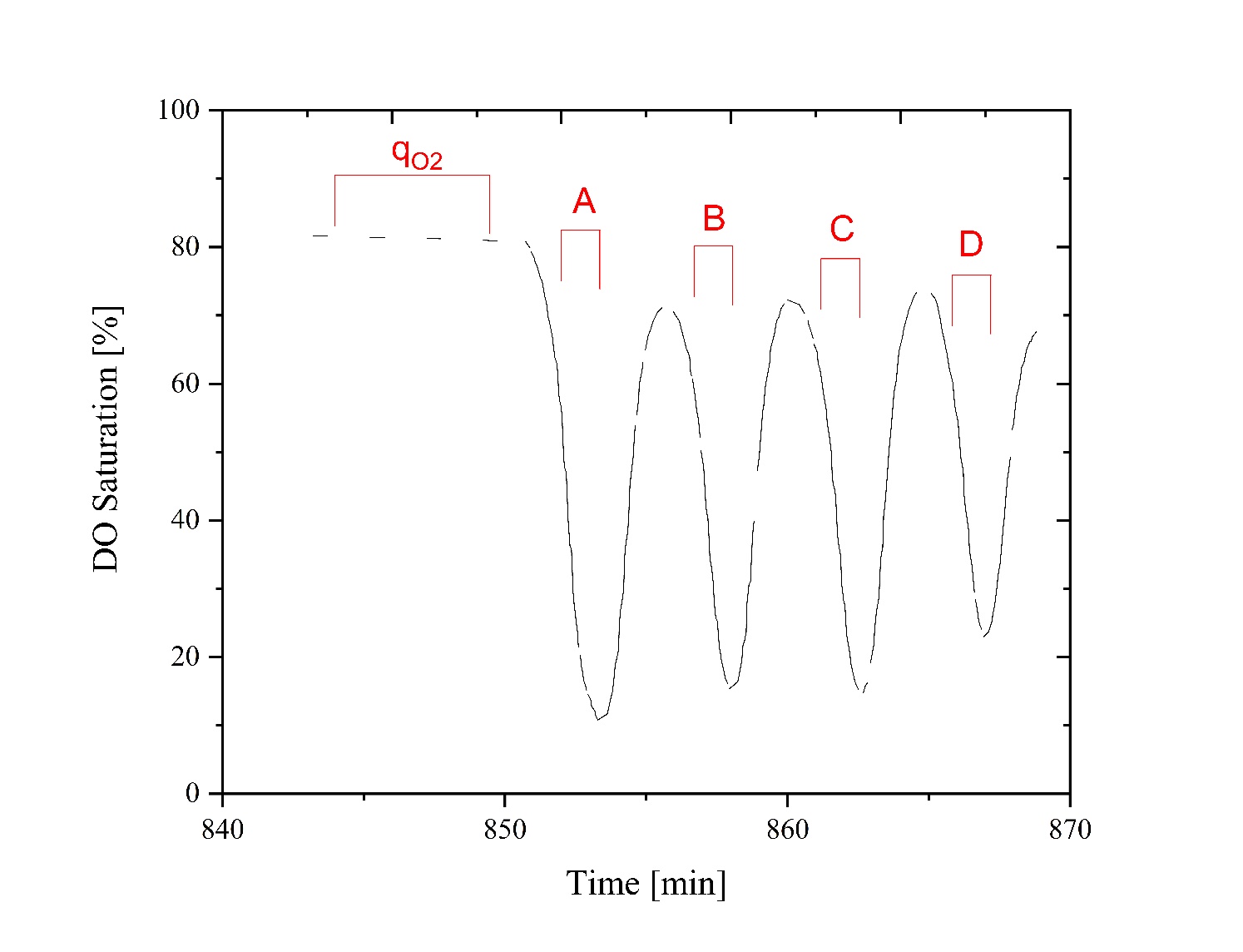


Supplementary Figure 4: DO saturation curve during the fermentation of *K. lactis*. The curve was recorded during fermentation with an aeration rate of 1 vvm air and an agitation rate of 1000 rpm in a working volume of 3.43 L: $\mathbf{q}_{\mathbf{O}_{\mathbf{2}}}$ indicates the range in which the $\bar{\mathbf{OUR}}$ value was recorded, A-D shows the test intervals in which $\mathbf{c}_{\mathbf{L}}^{\mathbf{*}}$ was recorded.

As shown in Supplementary Figure 4, the oxygen solubility was determined four times (A-D) during fermentation with *K. lactis*. For this purpose, the aeration was switched off and the saturation concentration was determined based on the decreasing DO value. The calculation is shown schematically in Supplementary Figure 5 and in the following derivation:

**
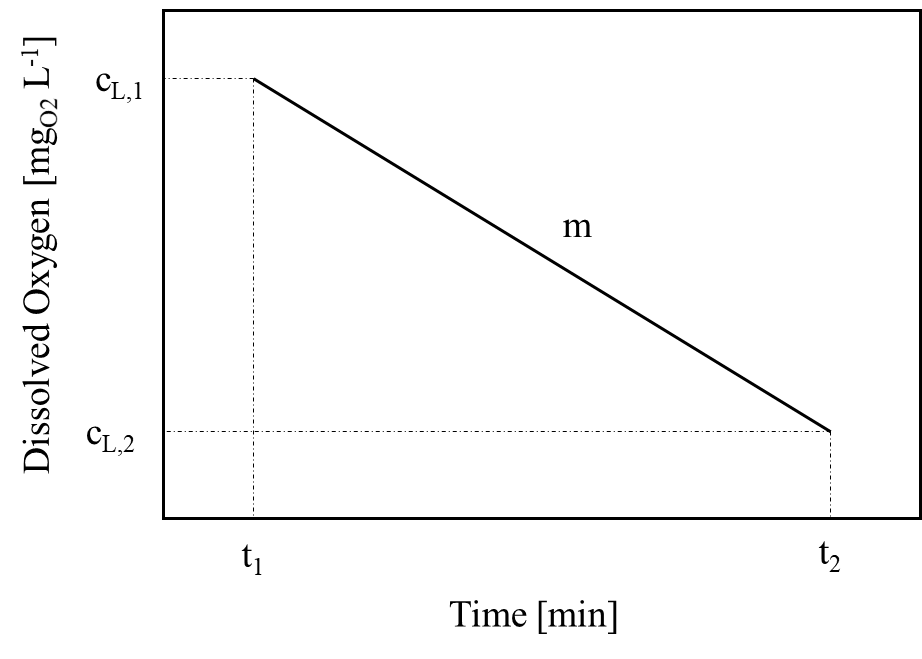
**

Supplementary Figure 5: Schematic DO response during determination of $\mathbf{c}_{\mathbf{L}}^{\mathbf{*}}\mathbf{.}$

The percentage DO can be expressed as the ratio of the DO concentration to the saturation concentration of oxygen in the medium, according to Supplementary Eq. 4.

$\%DO= \frac{c_{L}}{c_{L}^{*}} \times100\%$ **Supplementary Equation 4**

The decreasing DO follows a linear relationship. The y and x values (Supplementary Eq. 5) are displayed as intervals in which the linear decrease is valid. The slope (m) corresponds to the previously determined specific oxygen uptake ($q_{O_{2}}$) and the CDW from the corresponding test interval.

$\Delta y= -m \times\Delta x+b$ **Supplementary Equation 5**

where $\Delta y$ = c_L,2_-c_L,1_ $\left[ \frac{{mg}_{O_{2}}}{L} \right]$ and $\Delta x$ =$\Delta t$ = t_2_- t_1_ [min]. With $=q_{O_{2}}\times CDW$ $\left[ \frac{{mg}_{O_{2}}}{L \times min} \right]$; b = c_L,1_ ; c_L,1_ = $\frac{{DO}_{1}}{100\%}\times c_{L}^{*}\left[ \frac{{mg}_{O_{2}}}{L} \right]$ and c_L,2_ = $\frac{{DO}_{2}}{100\%}\times c_{L}^{*}\left[ \frac{{mg}_{O_{2}}}{L} \right]$ .

This leads to Supplementary Eq. 6:

$\frac{{DO}_{2}-{DO}_{1}}{100\%}\times c_{L}^{*}=-m \times\Delta t+ \frac{{DO}_{1}}{100\%} \times c_{L}^{*}$ **Supplementary Equation 6**

Division by $c^{*}$and rearrangement of the equation leads to Supplementary Eq. 7:

$c_{L}^{*}=\frac{-m \times\Delta t}{\frac{{DO}_{2}-{DO}_{1}-{DO}_{1}}{100\%}} \left[ \frac{{mg}_{O_{2}}}{L} \right]$ **Supplementary Equation 7**

Supplementary Table 1: Results of $\mathbf{c}_{\mathbf{L}}^{\mathbf{*}}$ during fermentation: $\mathbf{q}_{\mathbf{O}_{\mathbf{2}}}\mathbf{=3.59}\frac{\mathbf{mg}_{\mathbf{O}_{\mathbf{2}}}}{\mathbf{g}_{\mathbf{CDW}}\boldsymbol{\times min}}$ .

| Outgassing interval [-] | CDW [g L^-1^] | DO_1_ [%] | DO_2_ [%] | Δt [min] | $c_{L}^{*}$ $\left[ \frac{{mg}_{O_{2}}}{L} \right]$ |
| --- | --- | --- | --- | --- | --- |
| A | 2.55 | 43.2 | 31.5 | 0.40 | 6.67 |
| B | 2.61 | 60.7 | 37.8 | 0.58 | 6.50 |
| C | 2.69 | 67.6 | 48.5 | 0.58 | 6.46 |
| D | 2.75 | 56.3 | 38.0 | 0.40 | 5.29 |

Based on the results in Supplementary Table 1 the saturation concentration of oxygen was determined as 6.23 ± 0.63 mg_O2_ L^-1^.

# Literature

Vendruscolo, Francielo; Rossi, Márcio José; Schmidell, Willibaldo; Ninow, Jorge Luiz (2012): Determination of Oxygen Solubility in Liquid Media. In: *ISRN Chemical Engineering* 2012, S. 1–5. DOI: 10.5402/2012/601458.
